# Molecular Modeling Studies on the Multistep Reactivation Process of Organophosphate-Inhibited Acetylcholinesterase and Butyrylcholinesterase

**DOI:** 10.3390/biom11020169

**Published:** 2021-01-27

**Authors:** Jakub Jończyk, Jędrzej Kukułowicz, Kamil Łątka, Barbara Malawska, Young-Sik Jung, Kamil Musilek, Marek Bajda

**Affiliations:** 1 Department of Physicochemical Drug Analysis, Faculty of Pharmacy, Jagiellonian University Medical College, Medyczna 9, 30-688 Kraków, Poland; jakub.jonczyk@uj.edu.pl (J.J.); jedrzej.kukulowicz@doctoral.uj.edu.pl (J.K.); kamil.latka@doctoral.uj.edu.pl (K.Ł.); mfmalaws@cyf-kr.edu.pl (B.M.); 2 Division of Bio and Drug Discovery, Korea Research Institute of Chemical Technology, Daejeon 34114, Korea; ysjung@krict.re.kr; 3 Department of Medicinal and Pharmaceutical Chemistry, University of Science and Technology, Daejeon 34113, Korea; 4 Department of Chemistry, Faculty of Science, University of Hradec Kralove, Rokitanskeho 62, 500 03 Hradec Kralove, Czech Republic; 5 Biomedical Research Center, University Hospital in Hradec Kralove, Sokolska 581, 500 05 Hradec Kralove, Czech Republic

**Keywords:** molecular modeling, reactivators, reactivation process, organophosphates, docking studies, acetylcholinesterase, butyrylcholinesterase

## Abstract

Poisoning with organophosphorus compounds used as pesticides or misused as chemical weapons remains a serious threat to human health and life. Their toxic effects result from irreversible blockade of the enzymes acetylcholinesterase and butyrylcholinesterase, which causes overstimulation of the cholinergic system and often leads to serious injury or death. Treatment of organophosphorus poisoning involves, among other strategies, the administration of oxime compounds. Oximes reactivate cholinesterases by breaking the covalent bond between the serine residue from the enzyme active site and the phosphorus atom of the organophosphorus compound. Although the general mechanism of reactivation has been known for years, the exact molecular aspects determining the efficiency and selectivity of individual oximes are still not clear. This hinders the development of new active compounds. In our research, using relatively simple and widely available molecular docking methods, we investigated the reactivation of acetyl- and butyrylcholinesterase blocked by sarin and tabun. For the selected oximes, their binding modes at each step of the reactivation process were identified. Amino acids essential for effective reactivation and those responsible for the selectivity of individual oximes against inhibited acetyl- and butyrylcholinesterase were identified. This research broadens the knowledge about cholinesterase reactivation and demonstrates the usefulness of molecular docking in the study of this process. The presented observations and methods can be used in the future to support the search for new effective reactivators.

## 1. Introduction

Organophosphorus compounds (OPs) have been known for years as effective pesticides used in agriculture (e.g., paraoxon and chlorpyriphos) and as chemical weapons (e.g., sarin, venomous agent X (VX), tabun, and soman) belonging to the so-called “nerve agent” class representing some of the strongest man-made poisons [1,2,3]. The toxic effects of OPs result from irreversible blockade of acetylcholinesterase (AChE), the enzyme responsible for the degradation of acetylcholine [1,3]. The accumulation of this neurotransmitter in cholinergic synapses within the central and peripheral nervous system causes, among other effects, muscle tremors, spasms, cardiac dysfunction, and respiratory failure, often leading to death [4,5]. In addition to blocking AChE, OPs also have the ability to inhibit butyrylcholinesterase (BuChE), which also degrades other choline esters in addition to acetylcholine. The World Health Organization (WHO) estimates that approximately 3 million people are accidentally or intentionally intoxicated each year by OPs used as pesticides, with approximately 220,000 of them being poisoned fatally [6]. Although usage of chemical weapons is internationally banned, there are unfortunately still cases of the deliberate use of OPs, such as the terrorist attacks in the Tokyo subway in 1995, the bombing of civilians in Syria from 2013 to date, the use of a Novichok agent against Sergei and Yulia Skripal in 2018, and the recent poisoning of Alexei Navalny [7,8,9]. Treatment options for OP intoxication are limited. They are based on the administration of atropine, which reduces the overstimulation of muscarinic receptors, benzodiazepines that reduce convulsions, and oximes, which are specific antidotes capable of reactivating AChE [5,10]. The effectiveness of oximes depends on the type of OP and the time elapsed from poisoning. The small number of such compounds currently available for use as drugs and their often low activity result in the need to search for new reactivators. Intravenous administration of BuChE, alone or in combination with its reactivator, as a pseudocatalytic bioscavenger of OPs in the bloodstream is being considered for use as a supportive treatment that allows more rapid clearance of OPs from the patient [11,12]. However, the lack of effective BuChE reactivators currently limits the implementation of this therapeutic solution.

At the molecular level, the irreversible blockade of cholinesterases by OPs is based on the phosphorylation of serine, which is a part of the catalytic triad, also called the esteratic site, of the enzymes. This prevents the binding of the ester group of acetylcholine at this site and its further hydrolysis [1,13]. The other residues included in the catalytic triad are histidine and glutamic acid. The OP-enzyme complex may undergo an aging process. This process involves the dealkylation of an alkoxy moiety from the phosphorus atom of the OP. This process is clinically unfavorable because the aged complex is not susceptible to reactivation [14,15,16]. Other important structural elements of cholinesterases are the anionic site, which is responsible for binding the substrate quaternary ammonium group, and the acyl pocket, which binds the alkyl chains of acyl moieties. There is also an oxyanion hole that stabilizes the transition complex during the enzymatic reaction and a peripheral anionic site (PAS) located at the entry to the active gorge [17]. Notably, the active site in BuChE is significantly larger than that in AChE. This is due to the replacement of some of the aromatic amino acids within the acyl pocket, anionic site, and PAS in AChE with smaller aliphatic or even polar residues in BuChE. This translates into different substrate specificities of these enzymes as well as selective binding of inhibitors or reactivators [17,18].

**Figure 1:**
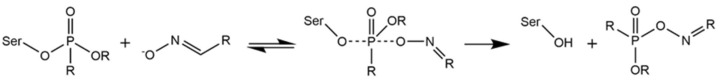
Mechanism of cholinesterase reactivation by oxime compounds.

The exact mechanism of cholinesterase reactivation by an oxime involves a nucleophilic attack of the oxime ion on the OP phosphorus atom bound to serine (SER) in the enzyme active site (Figure 1). As a result, a transient oxime-OP-SER adduct with a trigonal bipyramidal geometry is created. Subsequently, the OP-SER bond is broken, which restores the catalytic activity of cholinesterase and releases the OP-oxime complex. This complex should have low affinity for the binding enzyme pocket to quickly dissociate from it without rephosphorylation or further enzyme blockade [16,19,20].

**Figure 2:**
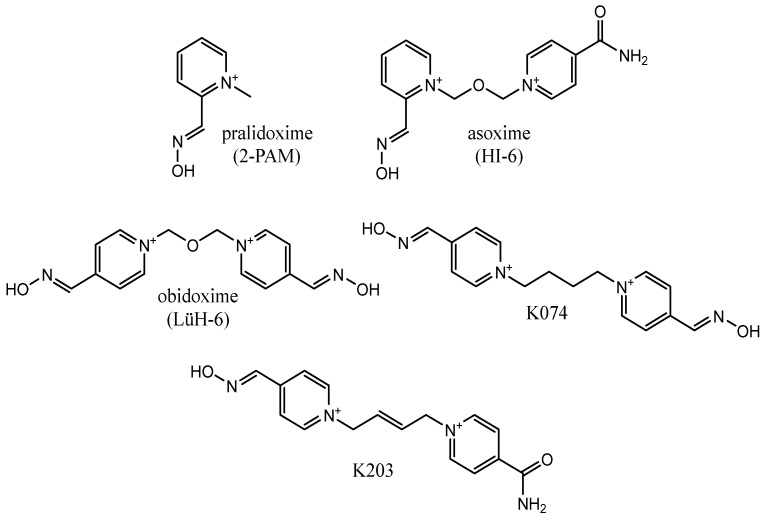
Structures of selected examples of cholinesterase reactivators.

Effective reactivators, in addition to a strong nucleophilic moiety such as the oxime moiety, must also have elements that allow interaction with the enzyme active site and facilitate favorable positioning of the oxime fragment with respect to the OP phosphorus atom. These criteria are fulfilled by the strongest reactivators currently known, which are quaternary pyridinium cations with an aldoxime moiety substituted in position 2 or 4 of the pyridine ring. Due to the number of aromatic rings in the structure, they can be divided into monopyridinium and bispyridinium derivatives [21]. Examples of such reactivators are presented in Figure 2 [21,22,23].

Although the crystal structures of both acetyl- and butyrylcholinesterase have been known for years, only some of them present a reactivator bound to the OP-enzyme complex. In addition, in almost all cases, the ligands have conformations in which the oxime moieties are located in the opposite direction from the OP fragment or are arranged in a geometrically unfavorable position that prevents nucleophilic attack on the OP phosphorus atom [24]. Allgardsson et al. [20] named this conformation nonreactive. They also obtained one crystal structure (PDB code: 5FPP) with the prereactivation conformation of the HI6 reactivator in chain A of the dimeric AChE complex inhibited by sarin. This structure presents the oxime moiety placed close to the phosphorus atom in a geometrically beneficial axial position. Interestingly, chain B obtained under the same conditions shows the nonreactivation conformation of asoxime (HI-6). It seems that the previous failures in the design of new effective reactivators may be partly caused by focusing only on the crystal structures that present reactivators in nonreactive conformations [25,26]. It is necessary to consider that an effective reactivator must be able to approach the phosphorus atom with the appropriate geometric constraints as presented by Allgardsson et al. [20]. Capturing this state by molecular modeling is not easy. It often requires the use of molecular dynamics techniques in addition to quantum methods because only they can reproduce the moment of oxime-OP bond formation [27,28,29,30]. Both molecular dynamics methods and, to an even greater extent, quantum calculations require high computational capacity, which limits their application.

In our work, we have studied the subsequent reactivation steps using relatively simple and fast molecular docking methods that are available to a wide group of researchers. In addition to the reactivation of acetylcholinesterase, we have also explored this process for butyrylcholinesterase. There is currently little research in the literature on this subject, although reactivation of BuChE appears to be beneficial from a pharmacological point of view. In addition, the presented studies consider an often overlooked state after reactivation when the OP fragment is bound to the oxime. The affinity of the oxime-OP complex to the cholinesterase active site is important for the efficiency of the reactivation process. The present research broadens the knowledge about the cholinesterase reactivation process and provides methods and observations that can support future design of new AChE and BuChE reactivators.

## 2. Materials and Methods

The three-dimensional ligand structures (free oximes and oxime-OP complexes) were built with the Corina online tool (Molecular Networks GmbH, Nürnberg, Germany and Altamira, LLC, Westport, CT, USA). Free oximes were prepared in the anionic form. Sybyl-X 1.1 (Certara USA, Princeton, NJ, USA) was used for the assignation of formal and partial charges with Gasteiger-Marsili and the preparation of mol2 files with proper atom types.

All proteins used in the docking studies were prepared with Hermes 1.7 (Cambridge Crystallographic Data Centre (CCDC), Cambridge, UK) [31]. All histidine residues were protonated at Nε, and the hydrogen atoms were added. All complexes with modified OP conformations (5FPP-like conformation in the prereactivation state) or with OP fragments transferred from other structures (all OP-BuChE complexes) were previously optimized with the protein preparation tool from Maestro Suite (Schrödinger, New York, NY, USA) [32].

All docking experiments were performed with the Gold v5.3 program (CCDC, Cambridge, UK) [31]. A standard set of genetic algorithm parameters with a population size of 100 and a number of operations of 100,000 was applied. The binding site was always defined as all amino acid residues within 15 Å from the oxygen atom from the hydroxyl group of serine 203 (AChE) or 198 (BuChE). As a result, 10 ligand conformations were obtained and sorted according to the values of the following scoring functions: the Astex Statistical Potential (ASP), GoldScore, ChemScore and ChemPLP. All results were visualized by PyMOL 0.99rc2 [33].

Docking studies were divided into four parts.

In the first stage of this study, presenting a nonreactive conformation of oximes, ligands were docked to complexes of AChE (2Y2V and 3DL4) and BuChE (3DJY with OP transferred from AChE complexes) inhibited by sarin and tabun. No constraints were assigned to the time of docking. The optimal docking parameters identified during the tests for this stage are summarized in Supplementary Materials Table S1.

In the second stage, the previously used protein complexes were modified so that the conformations of the OP present corresponded to the sarin conformation observed in chain A of 5FPP. Sarin-AChE and sarin-BuChE complexes were obtained by transferring the sarin-bound Ser203 directly from the 5FPP complex and subsequently optimizing the structures to remove potential steric clashes. In the case of tabun-AChE, the OP was first covalently docked to the empty 3DL7 active site. Subsequently, the pose with an ethoxy substituent bent in a similar way to that of the sarin isopropoxy group from 5FPP was selected and optimized. The tabun-BuChE complex was built by replacing fragments that distinguished both organophosphates while maintaining the positions of the overlapping heavy atoms based on the 5FPP complex as a template. After transferring a serine-tabun fragment, the entire complex was optimized. To map the prereactivation position of the pyridinium oxime observed in the reference protein, substructure-based constraints were added with the oxime moiety from cocrystallization structure of asoxime with AChE (5FPP chain A). The most favorable docking parameters are summarized in Supplementary Materials Table S2.

In the third stage, all prepared oxime-OP complexes with the phosphorus atom in the trigonal bipyramidal geometry were covalently docked to the serine of the catalytic triad of each cholinesterase (AChE—PDB code: 1J06 and BuChE—PDB code: 1P0I), recreating the bond between the oxygen and phosphorus atoms. Selected docking parameters are summarized in Supplementary Materials Table S3.

During the last stage of this study, the OP-oxime (POX) complexes representing the postreactivation state (phosphorus atom in tetrahedral geometry) were docked to AChE (PDB code: 1J06) and BuChE (PDB code: 1P0I). Docking parameters allowing to obtain the most coherent results at this stage of research are summarized in Supplementary Materials Table S4.

## 3. Results and Discussion

For our research, we chose tabun and sarin as model organophosphorus compounds. The processes of reactivation of AChE and BuChE were analyzed for the following selected oxime compounds: 2-PAM, HI-6, obidoxime, K074, and K203. The ability of these compounds to reactivate AChE and BuChE blocked by sarin and tabun is presented in Table 1.

**Figure 3:**
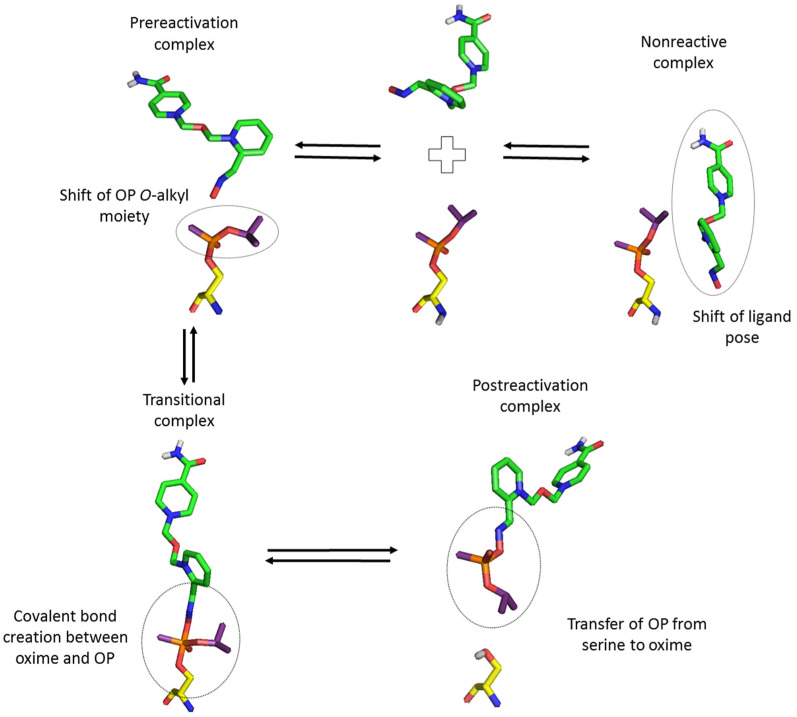
Multiple binding states during oxime reactivation of OP inhibited cholinesterase.

While modeling the reactivation process of cholinesterases blocked by organophosphorus compounds, we assumed the existence of several states in which the ligand could be found in the active site of the enzyme (Figure 3). Allgardsson et al. [19] proposed the presence of analogous states as part of the reactivation cycle. The crystal structure of AChE inhibited by sarin presented in their work shows a prereactivation state of HI-6 consistent with the mechanism of in-line attack of the reactivator on the phosphorus atom of an organophosphorus inhibitor [40]. As it is key for the reactivation process, we considered the balance between the formation of the presented complexes, which preferably should be shifted towards the stages immediately prior to reactivation. The energy differences between the prereactivation and transition complexes should be as small as possible, and the postreactivation OP-oxime complex should be poorly fitted to the active site. Moreover, to avoid additional enzyme blocking, the oxime should not show inhibitory effects. Free oximes were investigated as anions (monoanions in the case of obidoxime and K074) according to the reaction mechanism, indicating the direct participation of these forms in binding with OP. Poor dissociation of the oxime group was often indicated as the cause of the low activity of some reactivators in this analysis.

### 3.1. Nonreactive Complex

First, the nonreactive complex was analyzed. This complex is commonly observed in many crystals of AChE blocked by different OPs, e.g., in complexes stored under PDB codes: 5FPP (chain B), 5HFA, and 6CQV [20,41,42]. Our simulations also showed that the nonreactive complex is the conformation most readily achieved by ligands during docking to an active site blocked by OP. The analysis of this stage seems to be important both in the context of understanding the reasons for the behavior of the tested reactivators and to assess the preferences of individual ligands to adopt a prereactivation or nonreactive conformation. The information gained from this analysis will help guide further modification by shifting the balance between the two states in favor of the path leading to reactivation. To investigate the binding mode of the studied reactivators in a nonreactivating state, we docked them to the active site of AChE inhibited by sarin or tabun.

After docking to the sarin-AChE complex, most poses obtained for 2-PAM revealed conspicuous cation-π and π-π stacking interactions with Trp286 or Tyr341 (Supplementary Materials Figure S1). In the case of the tabun-AChE complex, the trend was completely different. Most of the results obtained for 2-PAM revealed a dominating arrangement in which Trp86 was involved in interactions with the pyridinium ring through π-π stacking or cation-π interactions.

Obidoxime revealed similar diversification in poses obtained for each AChE-OP complex (Supplementary Materials Figure S2). For AChE inhibited by sarin, the highest rated results showed the first pyridinium moiety interacting with Tyr124 (cation-π), Tyr337, and Tyr341 (CH-π). The other heterocyclic fragment was located in parallel with Trp286, allowing π-π stacking and cation-π interactions. AChE inhibited by tabun in most cases allowed obidoxime to interact with Trp86 by the pyridinium fragment in the parallel position while the other ring interacted with the PAS, most frequently with Trp286 and Tyr341.

**Figure 4:**
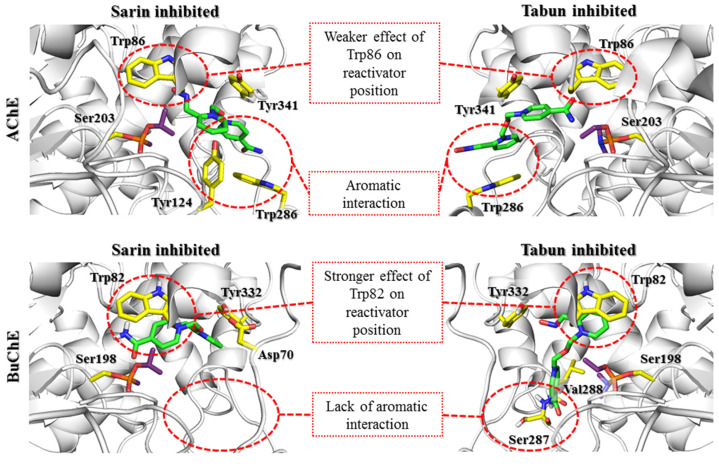
Docking results representing the nonreactivation conformation of the HI-6 (green) at the active site of AChE and BuChE (gray) blocked by sarin and tabun (purple). The key amino acids involved in the interactions with the reactivator are marked with yellow sticks. There is a clear difference in the binding of HI-6 in the active site of AChE and BuChE (the predominance of binding at the AChE PAS region and the BuChE anionic site).

Among the poses obtained for HI-6 during docking to sarin-AChE and tabun-AChE, we observed poses showing HI-6 with the amide group directed towards the catalytic site (Figure 4). A single pose for the sarin-AChE complex in a similar position to that from the 5FPP nonreactivated state was found. This dissimilarity resulted from the different conformations of Trp286, which, in our research, corresponded to the position observed in the unbound protein. Considering the above-mentioned single pose with an oxime fragment directed towards the interior of the active site of the enzyme, we observed an arrangement where the 1,2-disubstituted ring did not create any interactions, whereas oxygen belonging to the linker interacted with Tyr124 through hydrogen bond. The 1,4-disubstituted pyridinium ring interacted with Tyr341 (CH-π).

The results for K074 and K203 coincided with those of the corresponding bispyridinium compounds obidoxime and HI-6. In the case of K074 (Supplementary Materials Figure S3), the best rated pose for AChE inhibited by sarin was arranged such that Tyr124, Tyr337, and Tyr341 were engaged in interactions with the compound. The pyridinium ring located closer to the catalytic site interacted with Tyr124 through cation-π binding, while Tyr337 created a CH-π bond. Tyr341 interacted with both pyridine rings through CH-π interactions. K074 docking to tabun-inhibited AChE revealed a binding mode close to that of obidoxime docking to tabun-inhibited AChE; the first pyridinium ring interacted with Trp86 (CH-π), while the other interacted with Tyr341 (CH-π). K203 (Supplementary Materials Figure S4) in sarin-AChE was arranged analogically to the arrangement of HI-6 docking to sarin- and tabun-inhibited AChE and was arranged in a manner in which a 1,4-disubstituted ring with an amide group was directed towards the interior of the catalytic triad, while a 1,2-disubstituted pyridinium oxime was directed towards the exit of the narrow gorge. Only a single pose of K203 in complex with AChE inhibited by tabun revealed an arrangement where the aldoxime pyridinium remained at the catalytic site; for this pose, only a CH-π interaction with Tyr341 could be distinguished.

There are currently a limited number of complexes of OP-BuChE with reactivators available in the PDB database. The structure closest to the described nonreactive state is the 4AXB crystal structure, in which 2-PAM is bound to the aged soman-BuChE complex. In docking studies, oximes easily adopt a nonreactive arrangement. This indicates a high preference for the tested compounds to occur in this conformation. It is stabilized mainly by the π-π and cation-π interactions of the pyridinium oxime moiety with Trp82. In the case of bispyridinium compounds, the other aromatic fragment created similar interactions with Tyr332.

Detailed analysis of the docking results for individual compounds revealed a very high similarity of the obtained poses both in the complex with sarin-BuChE and tabun-BuChE. In the case of 2-PAM (Supplementary Materials Figure S1), there was a slight difference in the arrangement of the pyridinium ring that directed the methyl substituent in a different direction. However, it had no significant effect on the formation of cation-π and π-π interactions with Trp82 or hydrogen bonds with the hydroxyl group of Tyr332.

Obidoxime (Supplementary Materials Figure S2) in both complexes created cation-π and π-π interactions with Trp82 and Tyr332 and hydrogen bonds between the ionized oxime moiety and Tyr128. At physiological pH, the reactivator, which has two oxime groups, was mostly present in the form in which one group was dissociated and the other was not. This allowed for the creation of an additional hydrogen bond between the nonionized oxime group and the Pro285 backbone in the sarin-BuChE complex, and, in the case of tabun-BuChE, a hydrogen bond with the carboxyl group of Glu197 was observed.

The compound HI-6 showed the greatest differences in its arrangement depending on the OP blocking the active sites of BuChE (Figure 4). In docking to sarin-BuChE, the most common and highest rated arrangement was a pose in which the pyridinium ring connected to the amide group formed cation-π and π-π interactions with Trp82. The amide group formed a hydrogen bond with Glu197. The pyridinium oxime fragment interacted with Tyr332 and was involved in the ionic bond with Asp70. The position of the oxygen atom from the linker indicates the possibility of creating hydrogen bonds with Tyr332. In the case of docking the compound HI-6 into tabun-BuChE, the results were significantly different. The pyridinium moiety with oxime substituents formed cation-π and π-π interactions with Trp82, as with most of the other examined reactivators. The oxime group was involved in creating a hydrogen bond with the hydroxyl group of Tyr332. In turn, the amide group formed a network of hydrogen bonds with the Leu286 and Ser287 main chains.

The results for compounds K074 and K203 (Supplementary Materials Figures S3 and S4) were very similar to those described for the structurally related obidoxime and HI-6. K074 at the active site of BuChE blocked by sarin created cation-π and π-π interactions with Trp82 through the ionized pyridinium oxime group. The other aromatic fragment created a CH-π interaction with Tyr332, and the oxime connected to it formed hydrogen bonds with Thr120 and the main chain of Ser287. In the tabun-BuChE complex, compound K074 was placed closer to the catalytic triad; therefore, K074 was the only tested reactivator that could form aromatic interactions with both Trp82 and His438. As in the case of obidoxime, we also observed the formation of hydrogen bonds between the ionized oxime group and Tyr128. The other aromatic ring formed cation-π and π-π interactions with Tyr332 and ionic interactions with Asp70. Compound K203 in the sarin-BuChE complex, similar to HI-6, interacted with Trp82 via a pyridinium ring with an amide substituent. Probably due to the longer linker, this fragment can be located closer to the catalytic triad so that the amide group forms hydrogen bonds with both Tyr128 and Glu197. The other pyridinium ring formed cation-π and π-π interactions with Tyr332 as well as ionic bonds with Asp70, and the oxime moiety was directed towards Thr120, forming a hydrogen bond with it.

The docking results indicate that in both AChE and BuChE, the aromatic rings of tryptophan residues greatly affect the arrangement of reactivators in the active site. In the case of AChE, the influence of Trp286 in the PAS seems to be predominant. In our research, we tried to limit the influence of amino acids that change their conformation because there is no precise research on the kinetics of these changes over time or their impact on reactivator binding. The obtained results indicate the need for further studies shedding more light on the contribution of Trp286. The change observed in the sarin-AChE complex described by Allgardsson et al. [19] significantly facilitates the positioning of the bispyridinium reactivators such that the oxime group is directed towards the interior of the active site of the enzyme. However, in BuChE, as the PAS is reduced and lacks this residue, interaction with Trp82 within the anionic site appears crucial. The interactions with the PAS that dominate in AChE enable the formation of both prereactivation and nonreactive conformations. This indicates the ability to switch from one conformation to another. In turn, interactions with Trp82 in BuChE promote nonreactive conformations, which reduces the chance for effective reactivation of the enzyme.

### 3.2. Prereactivation Complex

The creation of a prereactivation complex is the first step in the process of reactivating serine blocked by an organophosphorus compound. This complex was experimentally confirmed in the 5FPP crystal structure (chain A) [19]. 5FPP represents HI-6 in the active site of sarin-inhibited AChE just before reactivation, in which the oxime group is directed towards the phosphorus atom, staying in close proximity. At the same time, this group remains in an axial relationship with the oxygen of Ser203. In addition to determining the position of the oxime at the moment of attack on the OP, a significant change in the position of the O-alkyl group is also observed. This change exposes the phosphorus atom, allowing the oxime anion to approach it.

To investigate this step of cholinesterase reactivation, modified crystal structures of AChE and BuChE inhibited by sarin or tabun were used. The modification concerned replacement of the original OP conformation with that known from 5FPP. This conformational change, which leads to exposure of the phosphorus atom to the attack of the oxime moiety, appears to be critical for obtaining the prereactivation state of the reactivator. During docking, we used constraints to obtain poses in which the oxime moiety is directed towards the OP phosphorus atom.

**Figure 5:**
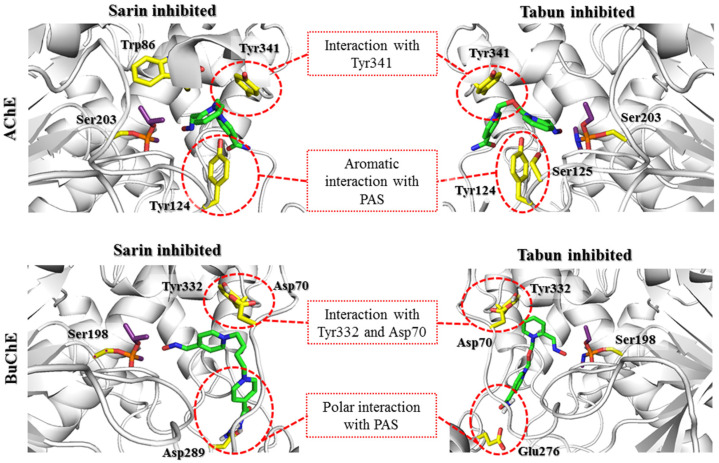
Docking results representing the prereactivation conformations of HI-6 (green) at the active sites of AChE and BuChE (gray) blocked by sarin and tabun (purple). The key amino acids involved in the interactions with the reactivator are marked with yellow sticks. The PAS region plays a huge role in ligand binding in both AChE and BuChE, despite the clear differences in the amino acids that build PAS of AChE and BuChE. Maintaining the BuChE prereactivation pose is dependent on the interaction with Tyr332 and Asp70. However, these interactions diminish during the formation of the transition state, in contrast to increasing interactions within the PAS AChE region.

Visual inspection of the results obtained by docking to sarin-AChE showed two noteworthy clusters of poses. In the first cluster, the location of the oxime group resembled the one known from 5FPP, whereas the second cluster showed an aldoxime pyridinium ring shifted towards Trp86, allowing for π-π or cation-π interactions. There were also poses in which the aldoxime pyridinium ring was located at an intermediate position between those from the first and second clusters. The second cluster represented poses in which the aldoxime group did not turn towards the phosphorus atom of sarin, as the HI-6 in 5FPP also created bad contacts with the isopropoxy substituent of sarin. Accordingly, with the assumption that the oxime group remains in an axial relationship with Ser203 oxygen, we further described poses that belonged to the abovementioned first cluster of poses. In the case of 2-PAM (Supplementary Materials Figure S5), the best rated pose created cation-π and π-π stacking with Tyr341 and hydrophobic contacts with the acyl pocket. The 1,2-disubstituted ring of HI-6 (Figure 5) was slightly shifted towards Trp86 but remained between the phenol groups of Tyr124 and Tyr337, allowing cation-π stacking. In turn, the 1,4-disubstituted pyridinium moiety was parallel to Tyr341. K074 (Supplementary Materials Figure S7) interacted with Tyr341 and the acyl pocket as observed for 2-PAM. The pyridinium oxime fragment not directed towards the phosphorus atom was able to interact with Trp286 through CH-π stacking. Additionally, its oxime group interacted with the main chain amide moiety of Leu76. K203 showed π-π stacking interactions of the aldoxime pyridinium with Tyr341 and Phe338. The pyridinium ring with an amide moiety remained parallel at a close distance to Trp286. The amide group interacted with Tyr72 through hydrogen bonds. The obidoxime-binding mode (Supplementary Materials Figure S6) was the same as that for K203 (Supplementary Materials Figure S8), excluding interactions with Tyr72.

In the case of docking of reactivators to the tabun-AChE complex, for all scoring functions, the lowest rated oxime was 2-PAM (Supplementary Materials Figure S5), which also has the lowest reactivation ability. The scoring function results revealed accordance between their values and the activities of the reactivators. Oximes were arranged such that the oxime group was in axial relation with the Ser203 oxygen atom. To gain insight into pose clusters, three pose arrangements were distinguished. The first represents the group of oximes shifted towards the oxyanion hole, the second represents those shifted towards His447 and Phe338, and the third represents the intermediate position between the first and the second. Visual assessment of 2-PAM allowed us to observe interactions with Tyr124 and Tyr337 through cation-π and π-π stacking, while the oxime group was directed towards the phosphorus atom of tabun. The results obtained for HI-6 docking showed significant differentiation of poses (Figure 5). Based on the best rated poses, the key interaction seems to be cation-π interaction of the 1,2-disubstituted pyridinium ring with Tyr124. Additional contacts were made by the 1,2-disubstituted ring with Ser125 through cation-π interactions and a 1,4-disubstituted pyridinium ring with Tyr341 through cation-π and π-π interactions. Docking of K074 (Supplementary Materials Figure S7) revealed consistent poses that represented one binding mode. For example, the best rated pose for one pyridinium ring was placed at the catalytic site, creating cation-π interactions with Tyr124, while the other aromatic fragment created π–π stacking interactions with Trp286 and Tyr341 at the edge of the narrow gorge. The ionized oxime moiety was turned towards the amide group of the main chain of Arg296. Poses obtained in docking of K203 (Supplementary Materials Figure S8) showed high cohesion representing the same binding mode. The best rated pose showed the cation-π interactions of oxime containing a pyridinium ring with Tyr124, while the second amide ring created cation-π and π-π stacking interactions with Trp286. Furthermore, the amide group interacted with the main chain of Ser293 and Phe295 through hydrogen bonds. The best rated poses for obidoxime (Supplementary Materials Figure S6) were arranged as previously described for K074. Molecular modeling of the prereactivation state proved that the interaction of the pyridinium moiety with Tyr124 of AChE was crucial.

After docking to BuChE, all the obtained prereactivation poses of the tested oximes created cation-π, π-π, or CH-π interactions with Tyr332. The ionic bond with Asp70 was also found in most results. Therefore, it can be assumed that both amino acids are crucial in stabilizing the pyridinium oxime reactivators in the prereactivation position. However, the structure of the PAS in BuChE is not favorable for interactions with the studied compounds in this conformation. A significant difference between AChE and BuChE is the number of aromatic amino acids located near the entrance to the enzyme. When comparing the sequences of these proteins, we noticed that the aromatic residues building the PAS in AChE were replaced by short aliphatic or even polar amino acids in BuChE. Based on the 5FPP crystal structure, it is clear that for bispyridinium reactivators, the interactions with Tyr70, Tyr121, and Trp279, which are located in the PAS, are important for binding with AChE. In BuChE, these amino acids correspond to Asn68, Gln119, and Ala277, respectively. This is the reason for the worse fit of these reactivators to BuChE in the prereactivation conformation. The prereactivation position of 2-PAM (Supplementary Materials Figure S5) was generally the same in both the sarin-BuChE and tabun-BuChE complexes. The only specific observed interactions were cation-π and CH-π interactions with Tyr332.

The prereactivation arrangement of obidoxime (Supplementary Materials Figure S6) was also very similar for both complexes. The pyridinium ring with the attached ionized oxime moiety creates cation-π interactions with Tyr332 and ionic bonds with Asp70. In turn, the other pyridinium oxime fragment forms a hydrogen bond with the Leu273 main chain through the OH moiety. The position of this group can also be stabilized by hydrogen bonds with Asn68.

The differences in the positions of compound HI-6 were more significant than those for the previously described reactivators. Although the pyridinium oxime fragment was placed near Tyr332, creating the interactions previously described for the other compounds, the further aromatic fragment exhibited a more diverse arrangement. It formed hydrogen bonds with Asn289 (sarin-BuChE) or Glu276 (tabun-BuChE) through an amide moiety (Figure 5).

The results for K074 (Supplementary Materials Figure S7) and K203 (Supplementary Materials Figure S8) were similar to those for HI-6. K074 and K203 exhibited a number of interactions with Tyr332 (cation-π) and Asp70 (ionic). The position of the second heterocyclic fragment did not show such consistency. Among the results for K074 in the sarin-BuChE complex, it was observed that this fragment was directed towards the entrance to the enzyme, where it formed a hydrogen bond with Gln71. Concerning docking to tabun-BuChE, the pyridinium moiety was located deeper and formed hydrogen bonds with Gln119 and Asn289. K203, with a double bond in the linker, had reduced conformational flexibility, which resulted in a less favorable adjustment to BuChE near the entrance to the enzyme. In the case of the sarin-BuChE complex, there was a hydrogen bond between the amide group of K203 and Asn289. In the tabun-BuChE complex, the pyridinium ring substituted by the amide moiety of K203 did not create any specific interactions.

In summary, it is worth noting the similarities in the arrangement of reactivators in the cholinesterase active sites for this step. In both AChE and BuChE, the constraint-forced position of the oxime moiety promoted ligand conformation in which the aromatic fragment not involved in binding with the OP was directed towards Trp86/Trp82 (AChE/BuChE) or, for most of the results, towards the entrance to the enzyme active site. The conserved Asp74/Asp70, Tyr341/Tyr332, and Phe338/Phe329 residues significantly facilitated the pyridinium oxime fragment to adopt a prereactivation conformation. In AChE, there were additional aromatic residues in this area that considerably reduced space and thus oriented the reactivator directly to the OP-blocked serine. The larger space of the binding site in BuChE resulted in a shift of the pyridinium oxime moiety away from the position optimal for reactivation. This indicates the need to expand this fragment of the compound to compensate for the absence of aromatic residues that restrict the arrangement of the ligand. This modification could help the reactivator accommodate the prereactivation conformation at the BuChE active site. Another observation concerns the binding of the aromatic fragment within the PAS. As previously mentioned, the PAS in BuChE is significantly reduced compared to that in AChE. As a result, the specific interactions between the aromatic fragments of the ligands and the PAS in BuChE are considerably weakened. The interactions are usually limited to hydrogen bonds formed by the amide or oxime moiety of the reactivator. The arrangement of this part of the ligand in BuChE often corresponds to that observed in AChE. However, in the case of the latter, the presence of additional aromatic residues, especially Trp286, that can adapt to the compounds stabilizes the beneficial conformation of the entire ligand. Therefore, it appears that to increase the efficiency of BuChE reactivators, the fragment of the compound not directly involved in the reactivation process needs to be optimized to better fit the reduced PAS in BuChE.

### 3.3. Transitional State

The application of covalent docking allowed us to reproduce the next step of the reactivation process, i.e., the formation of transitional serine-OP-oxime complex at the cholinesterase binding sites. This is the step with the highest energy, involving and it involves the spatial reorganization of the substitutes and the creation of a covalent bond between the phosphorus atom and the oxygen atom of the oxime group with the simultaneous breaking of the phosphorus-serine bond. The serine-OP fragment changes the geometry from tetrahedral to trigonal bipyramidal with both serine and oxime oxygen atoms in one axis [19,43]. For the purpose of this step, OP-oxime complexes with the appropriate geometry were prepared. Then, they were covalently docked to AChE and BuChE with the creation of a bond between the oxygen atom of the serine from the catalytic triad and the phosphorus atom of the OP. As in the previous steps, docking was conducted using the four scoring functions available in GOLD. The criteria for selecting the best conformation included both the coherence and the reliability of the mapped poses. As the correct poses were considered, those in which the free oxygen atom from the OP interacted with the oxyanion hole and the O-alkyl and alkyl substitutes (amino-alkyl in the case of tabun) were arranged similarly to those observed in known crystal complexes.

**Figure 6:**
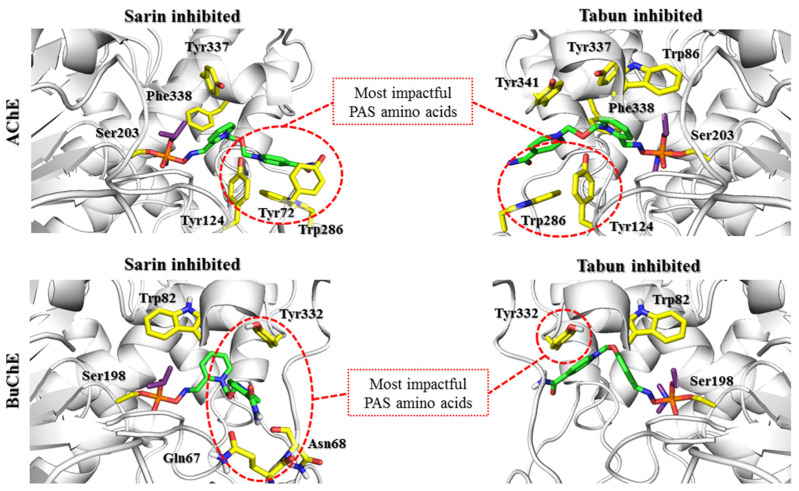
Docking results reflecting the formation of the transitional state by the HI-6 (green) at the active sites of AChE and BuChE (gray) blocked by sarin and tabun (purple). The key amino acids involved in the interactions with the reactivator are marked with yellow sticks. The interactions of the reactivator with AChE PAS region, observed in the prereactivation state, were further enhanced during the formation of the transitional state. In the case of BuChE, the weakening of the interactions with Tyr332 and Asp70 in the transitional state is not compensated by the PAS amino acids.

All the scoring functions enabled us to obtain the poses of the investigated oximes with attached sarin, in which the sarin binding mode was similar to that found in 5FPP. Regarding the pyridinium oxime fragment bound to sarin, the common feature of all conjugates regardless of scoring function was interaction with the phenol group of Tyr124. Additionally, this fragment interacted with Tyr341 and Phe338 through π-π stacking. The best scored pose for HI-6-sarin, in addition to interactions with Tyr341 and Phe338, was stabilized through cation-π and π-π stacking with Tyr337 (Figure 6). The best rated poses of K074, K203 and obidoxime revealed a binding mode similar to that of HI-6 (Supplementary Materials Figures S10 and S12). The pyridinium ring of these bispyridine compounds not bound to sarin mostly interacted with Trp286, creating cation-π and π-π stacking. Moreover, the amide groups of HI-6 and K203 were involved in hydrogen bonds with the phenol moiety of Tyr72.

The transitional complex for 2-PAM docked to tabun-AChE revealed the lowest values among the conjugates for all scoring functions (Supplementary Materials Figure S9). The values for the poses of the other oximes varied among all scoring functions and did not correlate with the experimental reactivation properties. Visual inspection of the best rated poses corresponding to the transitional state of 2-PAM-tabun showed a consistent binding mode of tabun that can be observed in original tabun-AChE complexes, for example, 3DL4. The phosphoryl oxygen interacted with the oxyanion hole through hydrogen bonds, whereas the dimethylamine group was directed towards the acyl pocket. Unlike the 3DL4 complex, the alkoxy substituent in this structure was bent in a manner similar to that of the sarin isopropoxy moiety found in 5FPP. The pyridinium ring neighbored Trp286, Tyr337, and Tyr124. The best rated pose showed cation-π interactions between the pyridinium moiety and Tyr337. Poses obtained for all scoring functions were coherent, but they differed from one another due to rotation changing affinities to neighboring residues. Covalent docking of HI-6-tabun to Ser203 had an effect similar to that of the earlier docking of 2-PAM-tabun regarding the arrangement of tabun (Figure 6). Most poses appeared in similar arrangements and represented the same binding mode. The 1,2-disubstituted pyridinium ring interacted with Trp86, Tyr337, and Phe338. The oxygen atom located at the linker fragment created a hydrogen bond with Tyr124. The 1,4-disubstituted pyridinium ring directed towards the exit of the narrow gorge was arranged between Trp286 and Tyr341. The amide group remained without any interactions.

The results obtained in the covalent docking of the tabun complex with K074 and K203 showed more varied poses (Supplementary Materials Figures S11 and S12). There were notable differences regarding the tabun arrangement, which was divided into two clusters. The first cluster was consistent with the previously described position regarding the 2-PAM-tabun and HI-6-tabun complexes, while in the other cluster, we observed phosphoryl oxygen directed towards Glu202. Regarding the proximal 1,4-disubstituted pyridine ring of these oximes, it is notable that it interacts with amino acids such as Trp86 and Tyr337 and with the phenol group of Tyr124. The best rated pose of K074 showed catalytic site interactions of the pyridinium ring with Tyr337 through T-shaped stacking while the other aromatic fragment interacted with Tyr341 through parallel π–π and cation-π stacking. The oxime group not linked with tabun did not show any significant bonding. The best rated result for K203 was arranged such that one pyridinium ring linked with the phosphorus atom created T-shaped π-π stacking with Tyr337. The other pyridinium ring was parallel to Tyr341, allowing π-π and cation-π interactions. The amide group remained unbound. The investigation of obidoxime (Supplementary Materials Figure S10) revealed interesting binding modes. The phenol group of Tyr124 simultaneously interacted with both pyridinium rings. Moreover, the aromatic fragment not directly bound to the OP remained at the PAS of AChE and interacted with Tyr341.

In the case of BuChE complexes, detailed analysis of the results showed some evolution of interactions characteristic of the prereactivation complex. The interactions with Tyr332 were crucial for stabilizing the position of all tested compounds; however, they were limited to cation-π interactions, with the exception of 2-PAM, which additionally created CH-π interactions. The movement of compounds deeper into the BuChE binding site hindered the formation of ionic bonds with Asp70. At the same time, it enabled the creation of cation-π and π-π interactions with Phe329 for most compounds. 2-PAM (Supplementary Materials Figure S9) shows the strongest interactions with Phe329 and Tyr332 in both the sarin-BuChE and tabun-BuChE complexes. The presence of equivalent amino acids in the AChE active site (Phe338 and Tyr341) might be one of the reasons for the very similar reactivation activity of 2-PAM towards AChE and BuChE.

Another analyzed compound, obidoxime (Supplementary Materials Figure S10), in addition to the previously described interactions with Phe329 and Tyr332, also created significant π-π stacking between the pyridinium oxime moiety not involved in binding the OP and Trp82 as well as a hydrogen bond between the nonionized oxime moiety and Glu197. This is a significant difference compared to the prereactivation pose, in which this pyridinium fragment was directed towards the entrance to the BuChE active site.

Compound HI-6, although similar in structure to obidoxime, did not obtain similar poses (Figure 6). The transitional complex with sarin-BuChE showed hydrogen bonds between the amide group of HI-6 and the Gln67 side chain as well as the Asn68 main chain. In turn, in a tabun-BuChE complex, asoxime was directed towards the entrance to the active site, creating a hydrogen bond with Tyr332. It is likely that changing the position of the oxime from 4- to 3- hindered the binding of the aromatic ring near Trp82.

K074 (Supplementary Materials Figure S11) in both the sarin- and tabun-blocked BuChE complexes created a transitional state in which a pyridinium oxime fragment not involved in binding the OP was directed towards the entrance to the enzyme, creating a hydrogen bond with the Gly283 main chain. The stiffening of the linker by introducing a double bond and replacing one oxime moiety with an amide group made the compound K203 the only one of the tested reactivators that did not form any specific interactions with Phe329 in any of the complexes. The K203 transitional state (Supplementary Materials Figure S12) in sarin-BuChE indicated additional cation-π interactions between the amide-bound pyridinium and Trp82. In the case of tabun-BuChE, this fragment was directed towards the entrance to the active site (in a pose analogous to that of compound K074), but no bonds that would stabilize this position were observed. It can be concluded that the ability of the pyridinium oxime moiety to create an optimal interaction with Phe329 and Tyr332 is key in the formation of the transitional complex by the studied reactivators. The substituents attached to the pyridinium ring not bound to OP should stabilize this position, as observed for obidoxime.

The results of covalent docking to the transitional state complexes of OP-AChE and OP-BuChE revealed further significant differences in the behavior of the tested reactivators. The highest rated poses for the 2-PAM transitional state with AChE and BuChE blocked by sarin or tabun presented a very similar position of the pyridinium fragment. This position created aromatic interactions with the Phe338/Phe329 and Tyr341/Tyr332 residues. In the case of AChE, an additional interaction with Tyr337 was observed. This is in accordance with the statement made in the previous step regarding the importance of interactions with these residues for the proper orientation of the pyridinium oxime fragment in a prereactivation state subsequently leading to a transitional state. Only the conformational changes of His447 and Phe338 present in the tabun-AChE complexes affected the position of the pyridinium ring, directing it towards Trp86. The results for the bispyridine compounds displayed high consistency of poses regarding docking to AChE and significant variation in the case of docking to BuChE. These discrepancies were again caused by the different structures of PAS in both enzymes. The aromatic residues of Tyr72 and Trp286 in AChE facilitated the arrangement of the aromatic fragment not directly bound to the OP within the PAS and thus positioned the entire ligand along the gorge of the enzyme. This binding mode was retained even for the 3DL4 complex, which exhibited the change in His447 and Phe338 conformations seen in tabun-AChE complexes. Moreover, the poses in which the ligand was placed over Phe338 clearly emphasize the limitations caused by this change in access to serine blocked by tabun. In the case of BuChE, the results can be divided into two main conformations. The first conformation is similar to that observed for AChE; however, in this case, the interaction with Tyr332 is crucial. The other conformation shows the U-shaped bend of the compound so that the pyridinium fragment not connected to the OP can interact with Trp82. It appears that the type of *O*-alkyl substituent of the OP affects the conformation of the ligand to some extent. In the case of a larger *O*-isopropyl fragment of the sarin, a smaller number of poses showed interactions with Trp82. In turn, for the *O*-ethyl fragment of the tabun, this number was higher. Knowing the difference in the efficiency of the tested reactivators in relation to cholinesterases blocked by tabun and sarin, it seems that the strength of ligand interaction with Trp86/Trp82 inversely correlates with their reactivation potency. This observation is reflected in the participation of this residue in the formation of the nonreactive conformation.

### 3.4. Postreactivation Complex

After breaking the bond with serine, the organophosphorus fragment is transferred to the reactivator, forming a stable complex. From the point of view of the reactivation process, the OP-oxime complex (POX) should not have affinity to the binding site [19]. This would indicate a low potential of such a complex acting as an enzyme inhibitor and allow for its rapid dissociation from the binding site. For the purpose of our research, we prepared a number of oxime-OP complexes that we then docked to the AChE and BuChE active sites to examine their arrangement and possible inhibitory potential.

**Figure 7:**
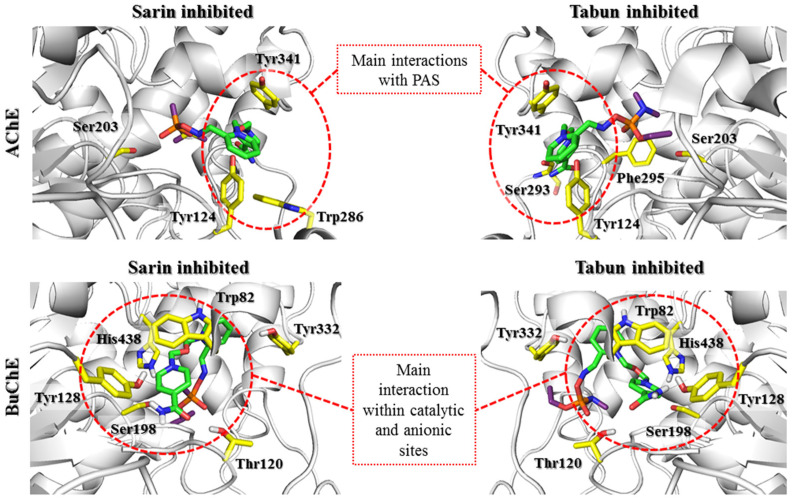
Docking results reflecting the behavior of the created sarin-HI-6 and tabun-HI-6 conglomerates (green) after reactivation of the active sites of AChE and BuChE (gray). The key amino acids involved in the interactions with the OP-reactivator conglomerate are marked with yellow sticks. The postreactivation complex binds more strongly at the active site of BuChE than AChE.

Among the poses obtained as a result of the docking of the sarin-oxime and tabun-oxime complexes to AChE, two clusters can be distinguished. In the first cluster, the poses were arranged such that the OP moiety remained at the catalytic site. The other cluster revealed an arrangement in which the pyridinium moiety not connected with the organophosphorus moiety was located at the catalytic site, whereas the sarin or tabun group was directed towards the outside of the narrow gorge. The best scored poses of the sarin-2-PAM conjugate belonged to the first cluster. Visual inspection showed that the phosphoryl oxygen atom of sarin was directed towards the oxyanion hole, while the pyridinium ring of 2-PAM (Supplementary Materials Figure S13) interacted with the aromatic ring of Tyr124 through cation-π and π-π stacking. The best rated results of sarin-HI-6 obtained in docking to AChE could be divided into two clusters. In the first cluster, we observed that the phosphoryl oxygen atom of sarin was adjacent to the oxyanion hole. However, the OP moiety did not create any interactions. The oxime component of the complex created cation-π and π-π stacking with Tyr341 and Trp286 via the pyridinium ring (Figure 7). Other sarin-oxime conjugates revealed coherent poses that belonged to the second cluster (Supplementary Materials Figures S14–S16).

Visual cluster analysis allowed us to notice that the organophosphorus group of tabun-oxime complexes with more active reactivators (K203, K074, and obidoxime) was preferably directed towards the exit of the narrow gorge, whereas for less potent oximes, it occurred more frequently at the catalytic site (Figure 7). The best rated pose in docking of tabun-2-PAM to AChE (Supplementary Materials Figure S13) showed an arrangement in which the phosphoryl oxygen of tabun was directed towards the oxyanion hole while the dimethylamine substituent was located near the acyl pocket. Ethoxy substituents occupied space at the exit of the catalytic pocket. The pyridinium ring of the investigated complex neighbored Tyr341 and Trp286 and also created cation-π interactions with the phenol group of Tyr124. In the case of tabun-asoxime complex, the best rated pose was arranged such that the OP moiety remained at the catalytic pocket without any significant interaction. However, the 1,2-disubstituted ring perpendicularly neighbored Tyr124, allowing for some π-π interactions. The phenol group of Tyr124 was directed towards HI-6, creating a hydrogen bond with an oxygen atom from the linker. The 1,4-disubstituted pyridinium ring was parallel to Tyr341 at a distance of 4–5 Å. The amide group of HI-6 was close to the main chains of Phe295 and Ser293, allowing hydrogen bonding. In the case of the tabun-K074 complex (Supplementary Materials Figure S15), the OP fragment remained in the catalytic site without any favorable interaction, while the 1,4-disubstituted ring attached to the OP fragment created cation-π stacking with the phenol group of Tyr124. Free aldoxime interacted with Gly342 through hydrogen bonds. Regarding the best rated pose for tabun-K203 (Supplementary Materials Figure S16), in which the OP component remained at the catalytic site, four interactions between pyridinium rings and AChE were noted. The aromatic fragment bound to the OP interacted with Tyr124 and Tyr341 via π-π stacking, while the phenol group of Tyr124 additionally interacted through cation-π stacking. The pyridinium ring with an amide moiety was parallel to Trp286 and allowed cation-π and π-π stacking. The OP component of the investigated conjugate did not create any significant interactions. The pyridinium fragment directly connected with the OP in the obidoxime-tabun complex (Supplementary Materials Figure S14) interacted with Tyr124 and Tyr341 by cation-π and π-π stacking. Furthermore, the close distance between the free aldoxime hydrogen atom and Ser293 main chain oxygen allowed hydrogen bonding.

The results of docking to BuChE obtained for the various scoring functions were very consistent, especially for the highest rated poses. The key amino acids for binding OP-oxime complexes could be easily identified. These include Trp82, which participated in the binding of all tested postreactivation complexes, and Tyr332, which stabilized the binding mode of compounds with two pyridinium fragments (obidoxime, HI-6, K074, and K203) (Figure 7).

A significant difference in the binding mode caused by the difference in the structure of the OP attached to the reactivator was observed for pralidoxime (Supplementary Materials Figure S13). For the sarin-pralidoxime complex, the pyridinium fragment was located among the aromatic rings of Trp82, Phe329, and Tyr332, creating hydrophobic and cation-π interactions. The oxygen atom of the sarin fragment additionally formed a hydrogen bond with Ser198 or His438. For the tabun-pralidoxime complex, in the most common pose, the pyridinium fragment was located between the Trp231 and Phe329 aromatic rings and also created hydrophobic and cation-π interactions. The oxygen atom of tabun was directed towards the oxyanion hole, but the ability to form hydrogen bonds in this position was limited.

Regarding the other reactivators, the differences caused by the type of attached OP were negligible. After binding to the OP, obidoxime (Supplementary Materials Figure S14) was arranged with a free pyridinium oxime fragment placed under the indole ring of Trp82, creating cation-π and π-π interactions. Nonreactive oxime group was in a convenient position to create hydrogen bonds with Tyr128 and Glu197. The sarin-bound pyridinium oxime fragment was placed parallel to the Tyr332 side chain, promoting cation-π and π-π interactions. This position was further stabilized by ionic bonds with Asp70. The sarin fragment facing the entrance to the active site created an additional hydrogen bond with Ser72.

The docking poses for the sarin-HI-6 and tabun-HI-6 complexes exhibited many interactions that were also observed in the previous obidoxime complexes (Figure 7). The amide-bound pyridinium ring formed cation-π and π-π interactions with Trp82. Additionally, the position of the ring allowed the creation of hydrogen bonds between the amide moiety and Thr120 and Tyr128. The main difference was the position of the sarin fragment, which was not directed towards the entrance to the active site of the enzyme but was located close to the catalytic triad. A hydrogen bond between the oxygen of the alkyloxy group and His438 was observed. A tabun fragment from an analogous complex was moved away from the catalytic triad; however, it was located deeper in the active site in this complex than in the tabun-obidoxime complex. The docking poses for the complexes of K074 and K203 (Supplementary Materials Figures S15 and S16) with both tabun and sarin were analogous to those observed for the docking of the obidoxime-OP complexes. The pyridinium ring substituted with an oxime (K074) or amide (K203) moiety was located parallel to the Trp82 side chain, forming cation-π and π-π interactions. This position was additionally stabilized by a hydrogen bond created between the oxime group and Tyr128. The second pyridinium ring was involved in cation-π and π-π interactions with Tyr332. Regardless of whether the compounds K074 and K203 formed complexes with sarin or tabun, the OP fragment was directed towards the entrance to the active site of BuChE, creating hydrogen bonds with the hydroxyl groups of the Ser72 and Gln71 main chains.

Comparing the obtained models representing the last stage of reactivation for AChE and BuChE, we observed that the differences in the trends of the arrangement of the compound depended on the structure of the reactivator and not on the type of OP or the particular cholinesterase. Both pralidoxime and HI-6 showed poor consistency of results with a slight predominance of poses in which the oxime-associated part of the postreactivation complex was directed inside the enzyme. On the other hand, reactivators such as obidoxime, K074, and K203 were arranged in a consistent manner in which the part associated with the OP was arranged within the outer part of the PAS. While the whole process of transition from the conformation in which the OP is located deep in the active site of a cholinesterase just after its bond with serine was broken to the conformation in which it is directed outwards requires further research on the molecular dynamics, it seems that stronger reactivators are characterized by this preference. It is also understood that the preference of the complex to leave the active site should positively correlate with the speed of this process and thus the restoration of the physiological functions of cholinesterase. The large number of poses in which we observed the OP-associated fragment facing outward tended to form an aromatic interaction of the pyridinium ring with Trp86/Trp82 so that the final pose was similar to the nonreactivating pose observed for the free reactivator docking.

## 4. Conclusions

In our study, we wanted to demonstrate the usefulness of a simple flexible molecular docking tool to model different steps in the reactivation process. We were pleased to find that by selecting various docking techniques, such as free flexible docking, flexible docking with distance constraints, and covalent docking, we obtained promising results that allowed us to conduct a comprehensive analysis of OP–reactivator–cholinesterase interactions. Many groups involved in research on the reactivation of cholinesterases focus on an important stage in the reactivation process: the moment when the reactivator adopts a prereactivation pose [20,41,44]. Based on the reactivation cycle presented by Allgardsson et al. [20] in which the reactivator can adopt nonreactivation or prereactivation conformations at the active site of OP-blocked cholinesterase, we performed an interaction analysis for five known reactivators modeling both states at AChE and BuChE active sites blocked by sarin or tabun. For both AChE and BuChE, the nonreactive conformation was reached by the reactivator more easily, which may suggest an equilibrium shift between these positions in favor of a nonreactivating position. Analysis of interactions allowed us to identify the amino acids Phe338/Phe329 and Tyr341/Tyr332, which occur in both AChE and BuChE, as key to obtaining the prereactivation conformation. Many teams using the MD and QM/MM methods in their research have also reached similar conclusions [29,45]. Our results show that the amino acids composing the PAS of AChE are an important factor facilitating the prereactivation conformation of reactivators of this enzyme. Differences between AChE and BuChE in the structure of this fragment are the reason for the significantly lower reactivation efficacy of the tested reactivators for BuChE. The results we obtained are in line with the observations of other teams and the mapped analogous arrangements observed in the complexes available in PDB. Vyacheslav et al. [46], using the MD approach on the phosphorylated enzyme-reactivator complex, compared simulations starting from prereactivation and nonreactive positions and showed that the prereactivation (apical) position of the studied reactivators was rather stable and thus maintained over the MD trajectory, while in the nonreactive (side) position, the hydroxamic group left the active site shortly after the beginning of the simulation. Unfortunately, the small number of complexes representing some of the stages presented here, especially the prereactivation, transient and postreactivation states, makes it difficult to refer to the experimental data. This was especially troublesome when developing models for BuChE. Our study is one of the few that have conducted a comprehensive analysis of the course of reactivation of BuChE blocked by an OP. On the one hand, this limits our ability to refine the method based on the results published so far, but it is an important step towards explaining the reasons for the high resistance of OP-inhibited BuChE to known reactivators. The observed differences in the positions of the analyzed reactivators in the active sites of AChE and BuChE and the numerous similarities constitute a good starting point for proposing structural modifications to the known reactivators. Based on our observations, it seems that to improve the effectiveness of reactivators for BuChE, the key is to introduce groups that will stabilize the molecule in the PAS in a manner analogous to the interactions observed in AChE. This change could be achieved by introducing moieties that interact with the amino acids forming the acyl loop.

In summary, we believe that the protocol we have presented allows rapid and effective analysis of many important stages of the reactivation process. The presented approach may be part of advanced algorithms utilizing quantum mechanics/molecular mechanics (QM/MM) or molecular dynamics (MD) methods. After optimization of the ligand assessment process, the described method can provide an independent screening tool for predicting the potential efficacy of reactivation by new compounds.

## Figures and Tables

**Table 1:** The kinetics of reactivation of sarin or tabun inhibited AChE and BuChE by the investigated oximes [34,35,36,37,38,39].

Cpd.	Sarin-AChE		Tabun-AChE		Sarin-BuChE		Tabun-BuChE	
	K_D_ (μM)	k_r2_ (min^−1^ M^−1^)	K_D_ (μM)	k_r2_ (min^−1^ M^−1^)	K_D_ (μM)	k_r2_ (min^−1^ M^−1^)	K_D_ (μM)	k_r2_ (min^−1^ M^−1^)
**Pralidoxime**	28.0	9300	3200	3.2	110	1500	1300	0.9
**Obidoxime**	31.0	30,000	1400	42.0	92.0	3500	-	-
**Asoxime**	50.0	14,000	-	-	1.1	18,000	1800	0.9
**K074**	-	-	2000	40.0	-	-	600	13.0
**K203**	-	-	56.0	2100	-	-	1900	4.0

K_D_—phosphylated enzyme-oxime dissociation constant; k_r2_—second-order reactivation rate constant.

## Data Availability

Not applicable.

## Supplementary Materials

The following are available online at https://www.mdpi.com/2218-273X/11/2/169/s1, Table S1: Detailed information about the docking parameters for Nonreactivating Conformation—Free Docking, Table S2: Detailed information about the docking parameters for Prereactivation Step—Docking with Substructure Based Constraints (Oxime Moiety from Chain A of 5FPP Complex, Table S3: Detailed information about the docking parameters for Transition State—Covalent Docking, Table S4: Detailed information about the docking parameters for Postreactivation State—Free Docking, Figures S1–S16: Visualization of docking results showing nonreactivating (S1–S4), prereactivation (S5–S8), transitional (S9–S12) and postreactivation (S13,S14) poses for 2-PAM, obidoxym, K074 and K203 reactivators.
