# Supplementary material for: Molecular Modeling Studies on the Multistep Reactivation Process of Organophosphate-Inhibited Acetylcholinesterase and Butyrylcholinesterase
Source: Biomolecules. 2021 Jan 27;11(2):169. doi: 10.3390/biom11020169 (PMC7912477; doi:10.3390/biom11020169)
Supplement: Supplementary file 1 [file biomolecules-11-00169-s001.pdf]

biomolecules

MDPI

Supplementary Materials

Part A – Docking procedure details

Table S1. Detailed information about the docking parameters for Nonreactivating Conformation—Free Docking.

<table>
<tr><th>Investigated complex</th><th>PDB code of the complex used in simulation</th><th>Pralidoxime (2-PAM)</th><th>Obidoxime (LüH-6)</th><th>Asoxime (HI-6)</th><th>K074</th><th>K203</th></tr>
<tr><td>Sarin-AChE</td><td>2Y2V</td><td colspan="5">ASP*</td></tr>
<tr><td>Tabun-AChE</td><td>3DL4</td><td>ChemPLP#</td><td colspan="4">ChemScore*</td></tr>
<tr><td>Sarin-BuChE</td><td rowspan="2">3DJY with OP transferred from AChE complexes</td><td colspan="5" rowspan="2">GoldScore*</td></tr>
<tr><td>Tabun-BuChE</td></tr>
</table>

* Leading scoring function allowing to obtain correct results for the analyzed compounds; # The scoring function providing analogous but more consistent pose distribution for a specific ligand.

Table S2. Detailed information about the docking parameters for Prereactivation Step—Docking with Substructure Based Constraints (Oxime Moiety from Chain A of 5FPP Complex.

<table>
<tr><th>Investigated Complex</th><th>PDB Code of the Complex Used in Simulation</th><th>Pralidoxime (2-PAM)</th><th>Obidoxime (LüH-6)</th><th>Asoxime (HI-6)</th><th>K074</th><th>K203</th></tr>
<tr><td>Sarin-AChE</td><td>2Y2V with 5FFP-like conformation of OP</td><td colspan="5">ChemPLP*</td></tr>
<tr><td>Tabun-AChE</td><td>3DL4 with 5FFP-like conformation of OP</td><td>ASP*</td><td>ChemPLP#</td><td colspan="3">ASP*</td></tr>
<tr><td>Sarin-BuChE</td><td rowspan="2">3DJY with OP transferred from AChE complexes</td><td colspan="5" rowspan="2">GoldScore*</td></tr>
<tr><td>Tabun-BuChE</td></tr>
</table>

* Leading scoring function allowing to obtain correct results for the analyzed compounds; # The scoring function providing analogous but more consistent pose distribution for a specific ligand.

Table S3. Detailed information about the docking parameters for Transition State—Covalent Docking.

Investigated Complex	PDB Code of the Complex Used in Simulation	Pralidoxime (2-PAM)	Obidoxime (LüH-6)	Asoxime (HI-6)	K074	K203
Sarin-AChE<br>Tabun-AChE	1J06			ASP*		
Sarin-BuChE<br>Tabun-BuChE	1P0I			GoldScore*		

* Leading scoring function allowing to obtain correct results for the analyzed compounds.
# The scoring function providing analogous but more consistent pose distribution for a specific ligand.

Table S4. Detailed information about the docking parameters for Postreactivation State—Free Docking.

Investigated Complex	PDB Code of the Complex Used in Simulation	Pralidoxime (2-PAM)	Obidoxime (LüH-6)	Asoxime (HI-6)	K074	K203
Sarin-AChE	1J06			ASP*		
Tabun-AChE		ASP*	ChemPLP#	ASP*	GoldScore#	ASP*
Sarin-BuChE<br>Tabun-BuChE	1P0I			GoldScore*		

* Leading scoring function allowing to obtain correct results for the analyzed compounds.
# The scoring function providing analogous but more consistent pose distribution for a specific ligand.

Part B - Visualization of docking results showing nonreactivating (S1-S4), prereactivation (S5-B8), transi-tional (S9-S12) and postreactivation (S13-S14) poses for 2-PAM, obidoxym, K074 and K203 reac-tivators.

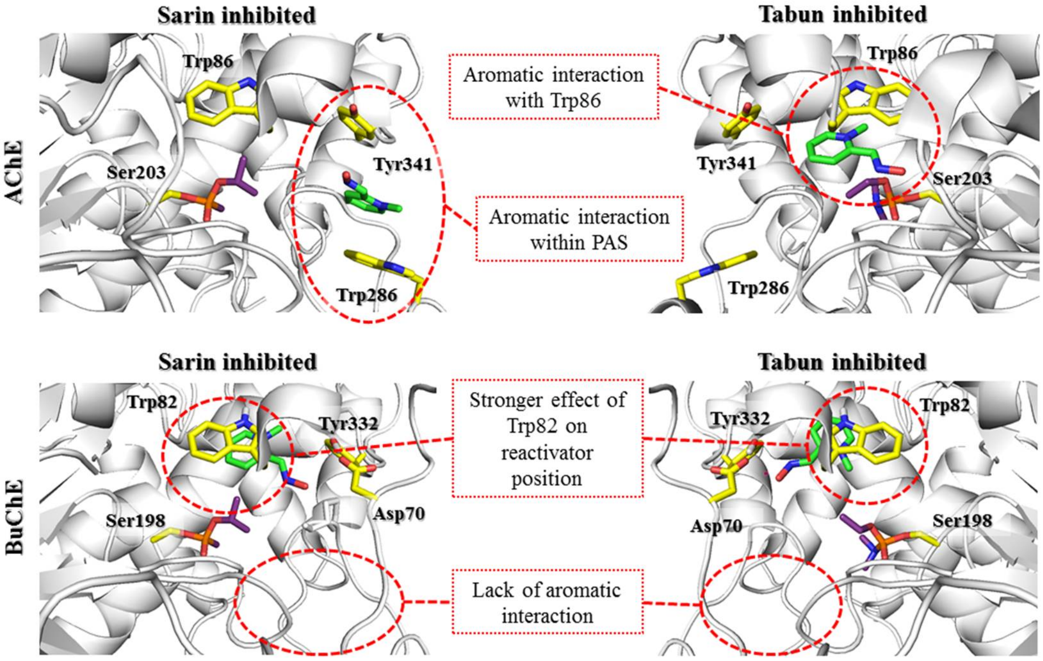

Figure S1. Docking results representing the nonreactivation conformation of the 2-PAM (green) at the active site of AChE and BuChE (gray) blocked by sarin and tabun (purple). The key amino acids involved in the interactions with the reactivator are marked with yellow sticks. 2-PAM binds nonreactively to AChE at two sites, analogously to the reactivator in PDB 5HFA complex.

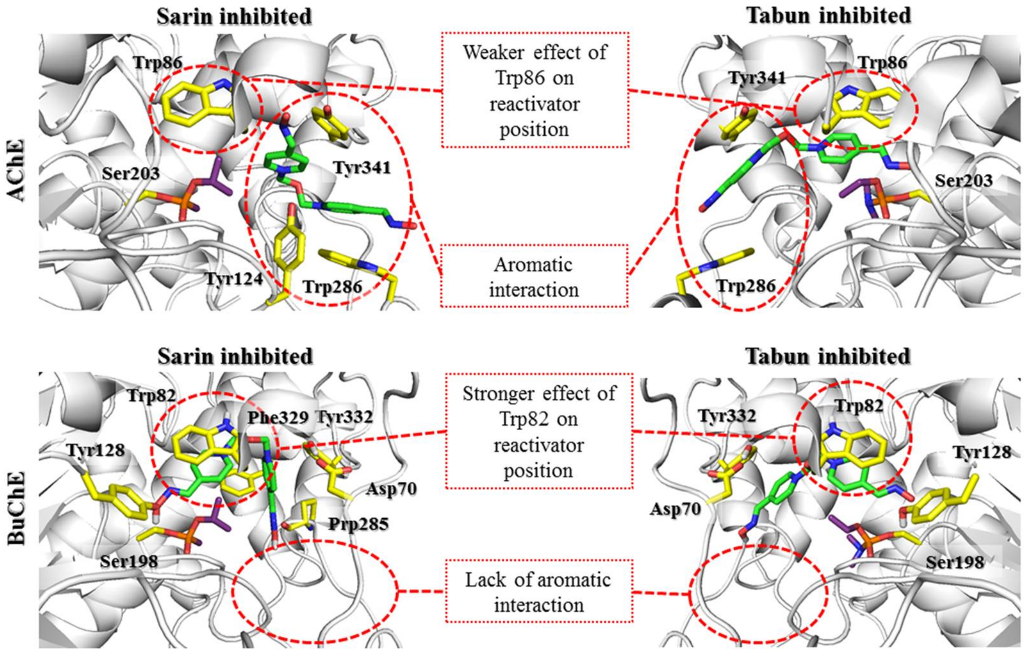

Figure S2. Docking results representing the nonreactivation conformation of the obidoxime (green) at the active site of AChE and BuChE (gray) blocked by sarin and tabun (purple). The key amino acids involved in the interactions with the reactivator are marked with yellow sticks. AChE

nonreactivation poses share a grip point within PAS with prereactivation poses. The binding to the Trp82 in BuChE stands in contrast to the creation of a prereactivation pose.

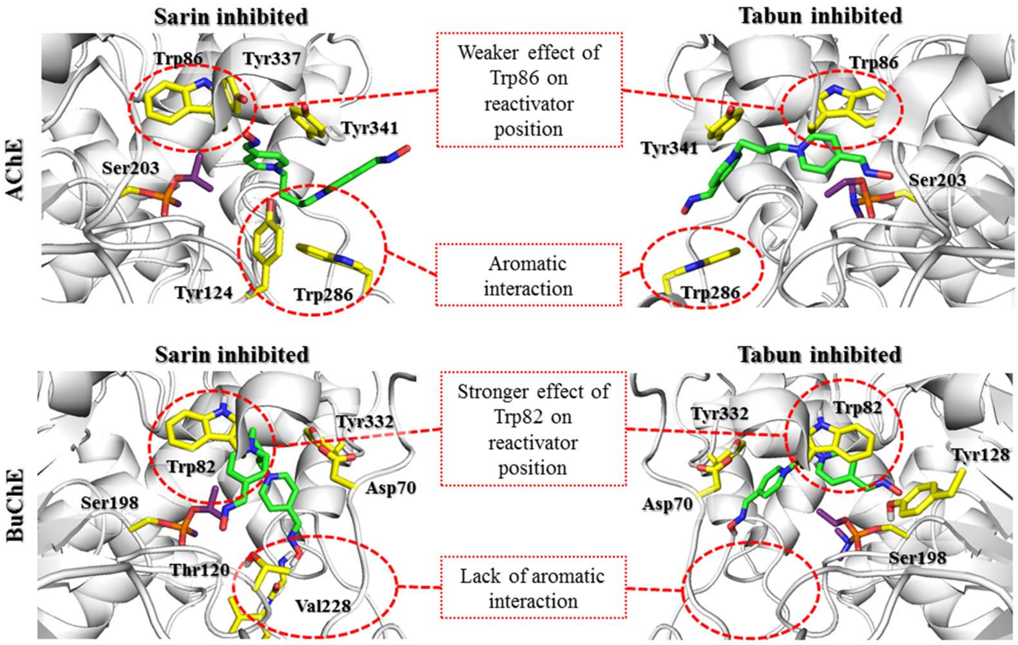

Figure S3. Docking results representing the nonreactivation conformation of the K074 (green) at the active site of AChE and BuChE (gray) blocked by sarin and tabun (purple). The key amino acids involved in the interactions with the reactivator are marked with yellow sticks. AChE nonreactivation poses share a grip point within PAS with prereactivation poses. The binding to the Trp82 in BuChE stands in contrast to the creation of a prereactivation pose.

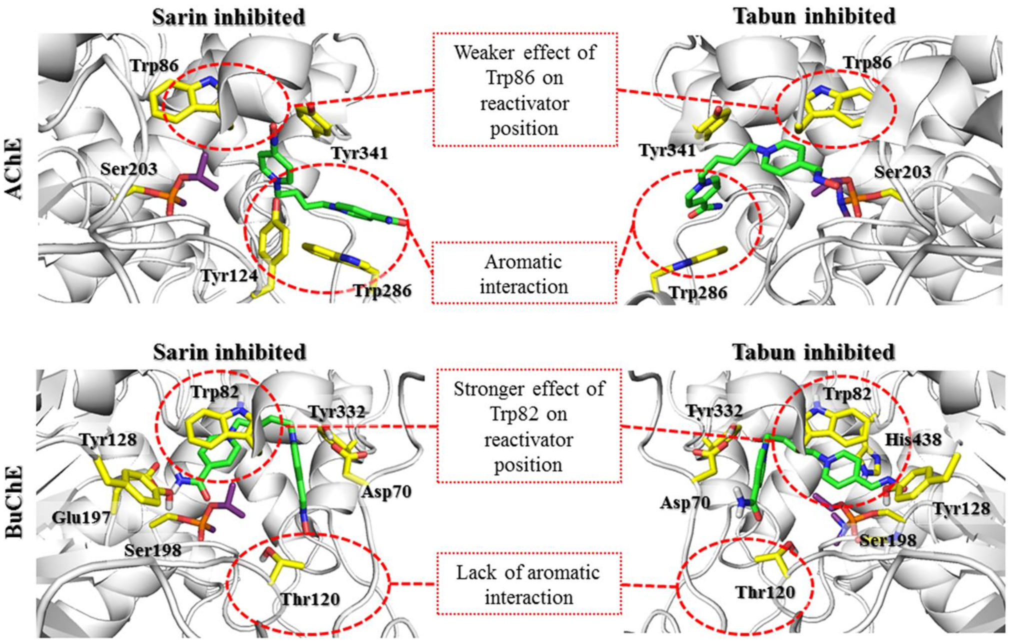

Figure S4. Docking results representing the nonreactivation conformation of the K203 (green) at the active site of AChE and BuChE (gray) blocked by sarin and tabun (purple). The key amino acids involved in the interactions with the reactivator are marked with yellow sticks. AChE nonreactivation poses share a grip point within PAS with prereactivation poses. The binding to the Trp82 in BuChE stands in contrast to the creation of a prereactivation pose.

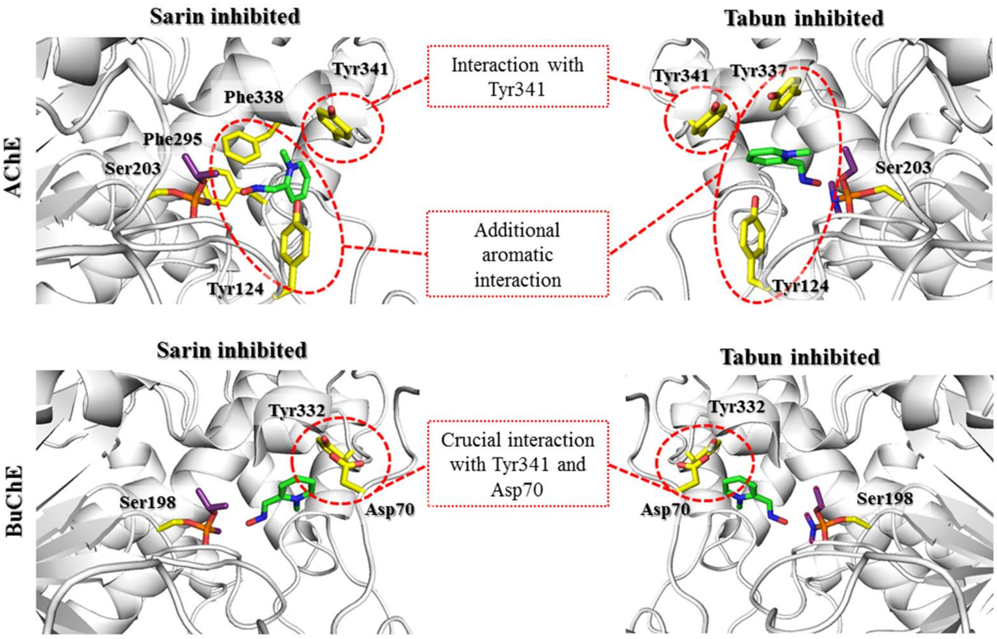

Figure S5. Docking results representing the prereactivation conformations of 2-PAM (green) at the active sites of AChE and BuChE (gray) blocked by sarin and tabun (purple). The key amino acids involved in the interactions with the reactivator are marked with yellow sticks. Maintaining the BuChE prereactivation pose is dependent on the interaction with Tyr332 and Asp70. However, these interactions diminish during the formation of the transition state, in contrast to increasing interactions within the PAS AChE region.

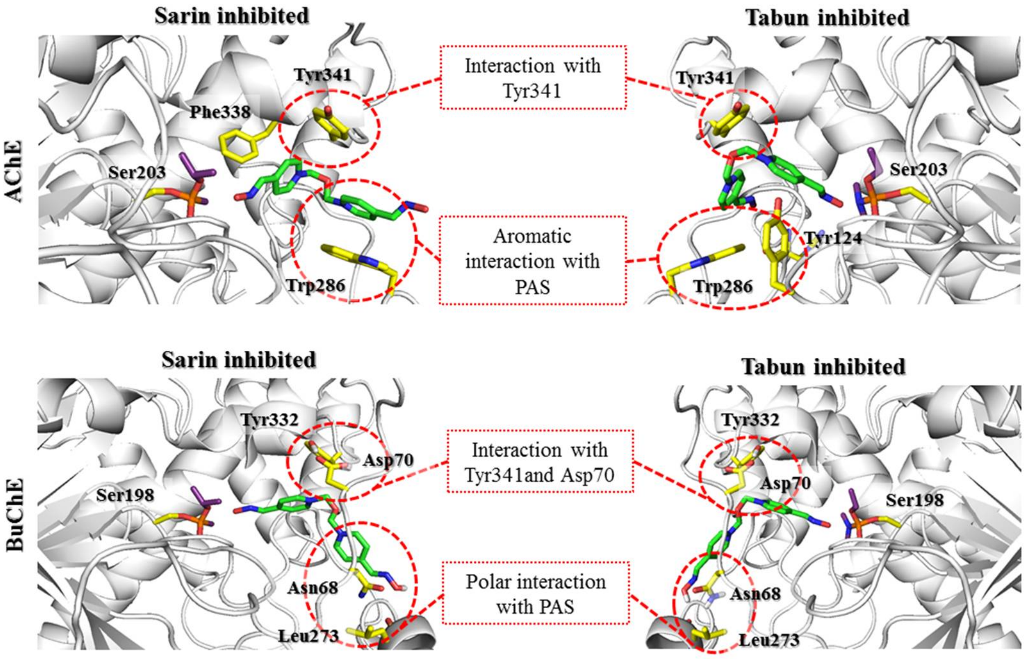

Figure S6. Docking results representing the prereactivation conformations of obidoxime (green) at the active sites of AChE and BuChE (gray) blocked by sarin and tabun (purple). The key amino acids involved in the interactions with the reactivator are marked with yellow sticks. Maintaining the BuChE prereactivation pose is dependent on the interaction with Tyr332 and Asp70. However, these interactions diminish during the formation of the transition state, in contrast to increasing interactions within the PAS AChE region.

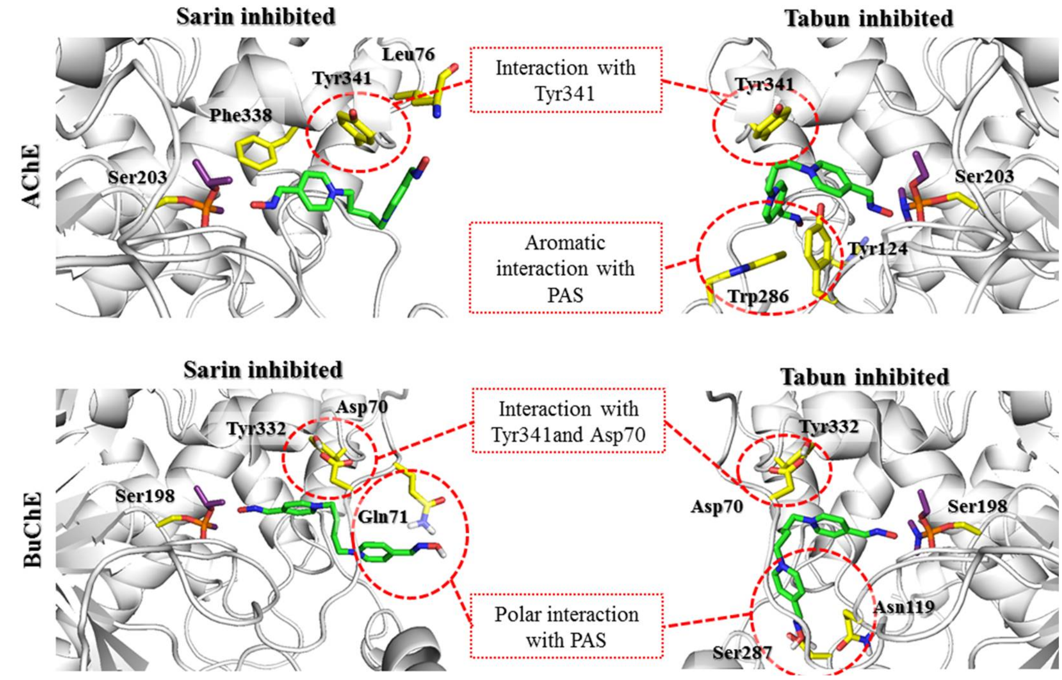

Figure S7. Docking results representing the prereactivation conformations of K074 (green) at the active sites of AChE and BuChE (gray) blocked by sarin and tabun (purple). The key amino acids involved in the interactions with the reactivator are marked with yellow sticks. Maintaining the BuChE prereactivation pose is dependent on the interaction with Tyr332 and Asp70. However, these interactions diminish during the formation of the transition state, in contrast to increasing interactions within the PAS AChE region.

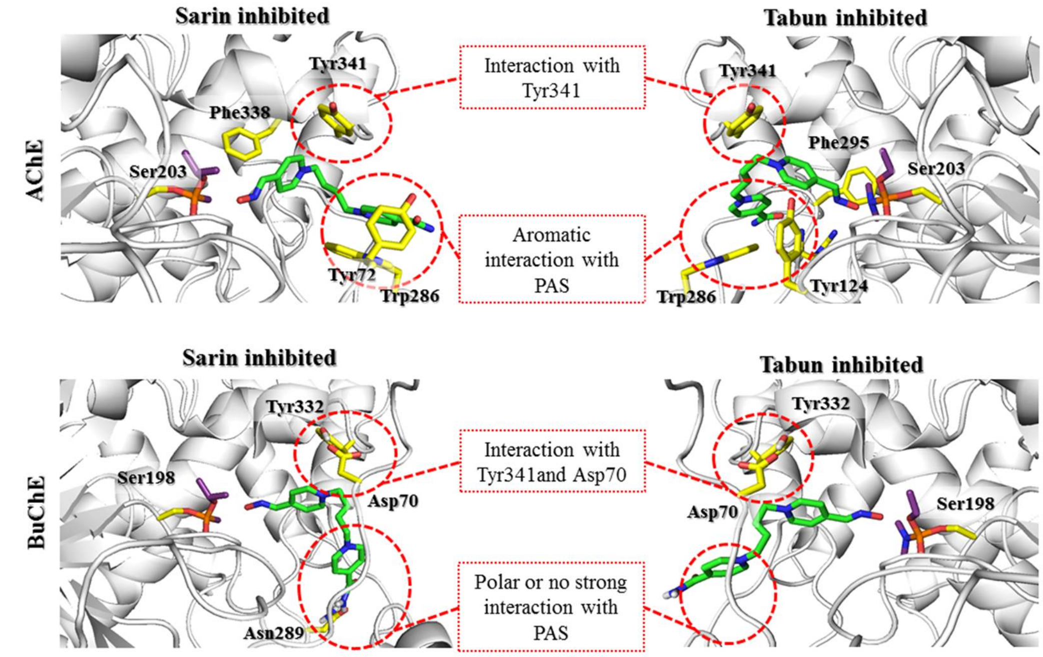

Figure S8. Docking results representing the prereactivation conformations of K203 (green) at the active sites of AChE and BuChE (gray) blocked by sarin and tabun (purple). The key amino acids involved in the interactions with the reactivator are marked with yellow sticks. Maintaining the BuChE prereactivation pose is dependent on the interaction with Tyr332 and Asp70. However, these interactions diminish during the formation of the transition state, in contrast to increasing interactions within the PAS AChE region.

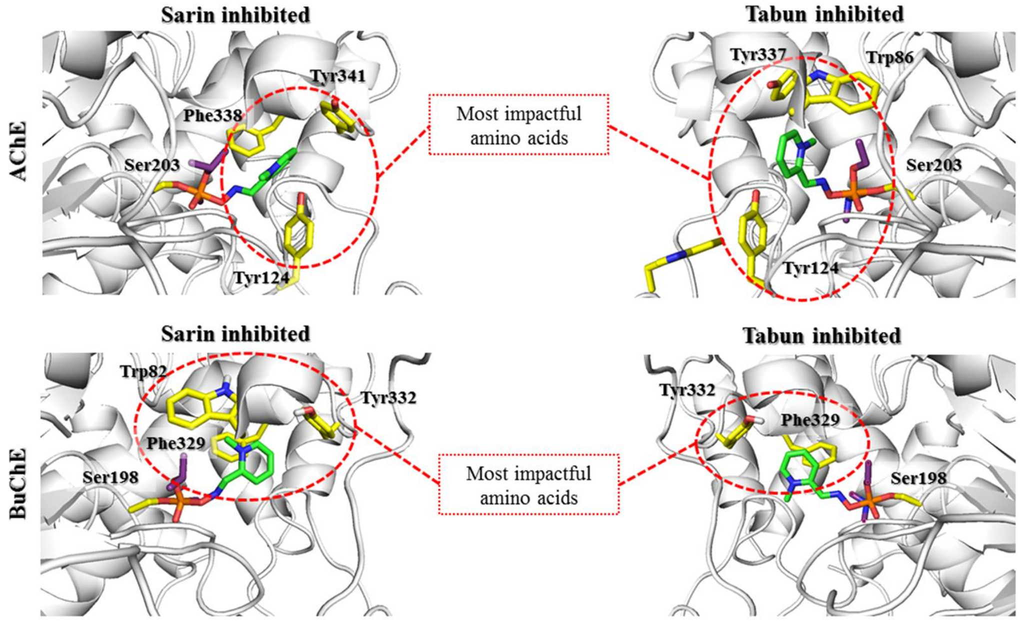

Figure S9. Docking results reflecting the formation of the transitional state by the 2-PAM (green) at the active sites of AChE and BuChE (gray) blocked by sarin and tabun (purple). The key amino acids involved in the interactions with the reactivator are marked with yellow sticks. The interactions of the reactivator with AChE PAS region, observed in the prereactivation state, were further enhanced during the formation of the transitional state. In the case of BuChE, the weakening of the interaction with Tyr332 and Asp70 in the transitional state is not compensated by the PAS amino acids.

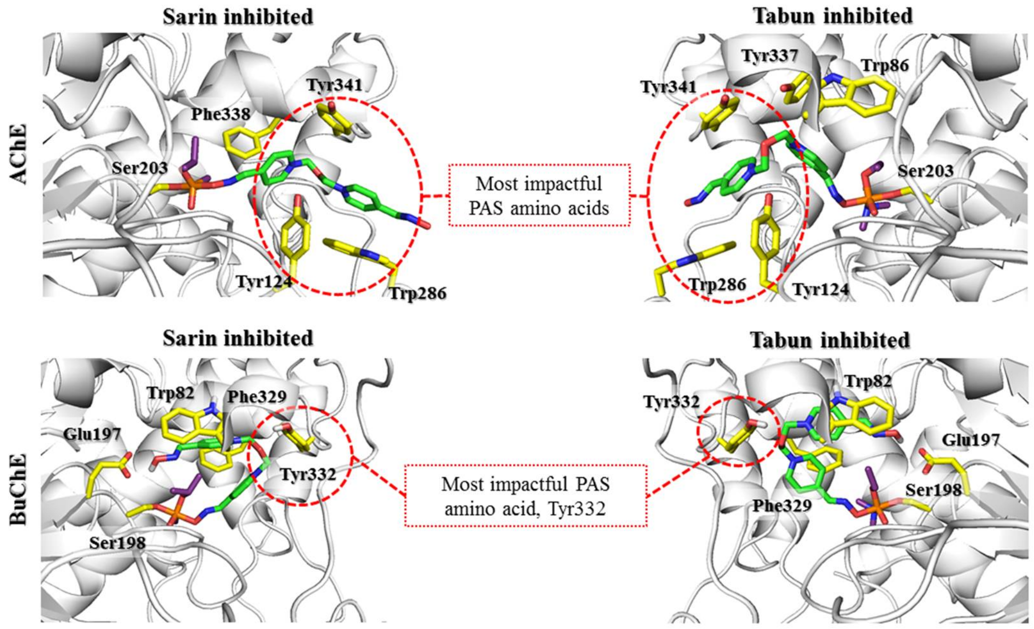

Figure S10. Docking results reflecting the formation of the transitional state by the obidoxime (green) at the active sites of AChE and BuChE (gray) blocked by sarin and tabun (purple). The key amino acids involved in the interactions with the reactivator are marked with yellow sticks. The interactions of the reactivator with AChE PAS region, observed in the prereactivation state, were further enhanced during the formation of the transitional state. In the case of BuChE, the weakening of the interaction with Tyr332 and Asp70 in the transitional state is not compensated by the PAS amino acids.

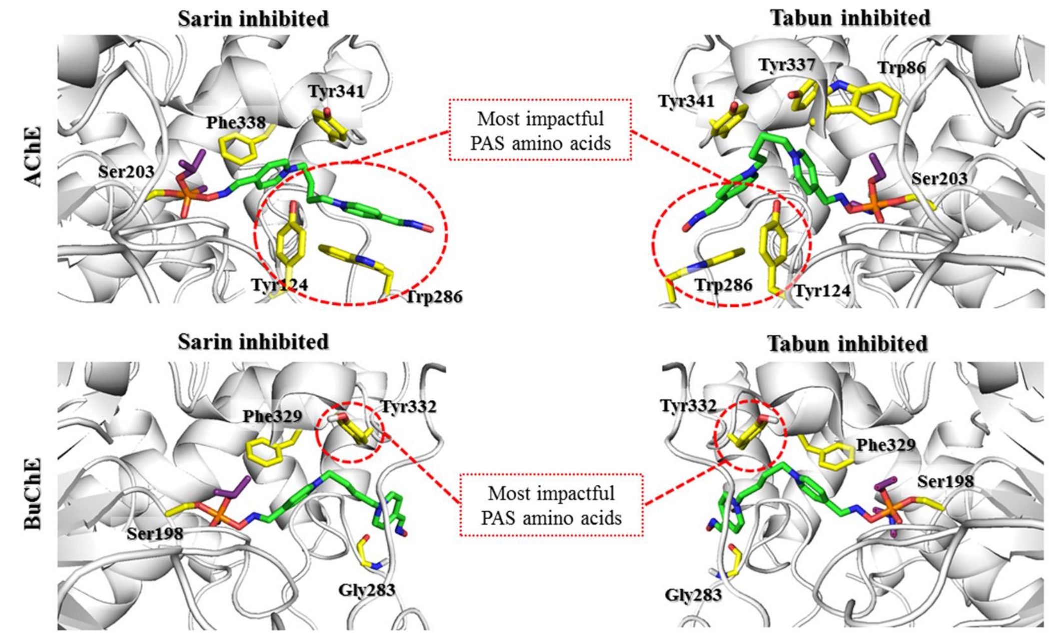

Figure S11. Docking results reflecting the formation of the transitional state by the K074 (green) at the active sites of AChE and BuChE (gray) blocked by sarin and tabun (purple). The key amino acids involved in the interactions with the reactivator are marked with yellow sticks. The interactions of the reactivator with AChE PAS region, observed in the prereactivation state, were further enhanced during the formation of the transitional state. In the case of BuChE, the weakening of the interaction with Tyr332 and Asp70 in the transitional state is not compensated by the PAS amino acids.

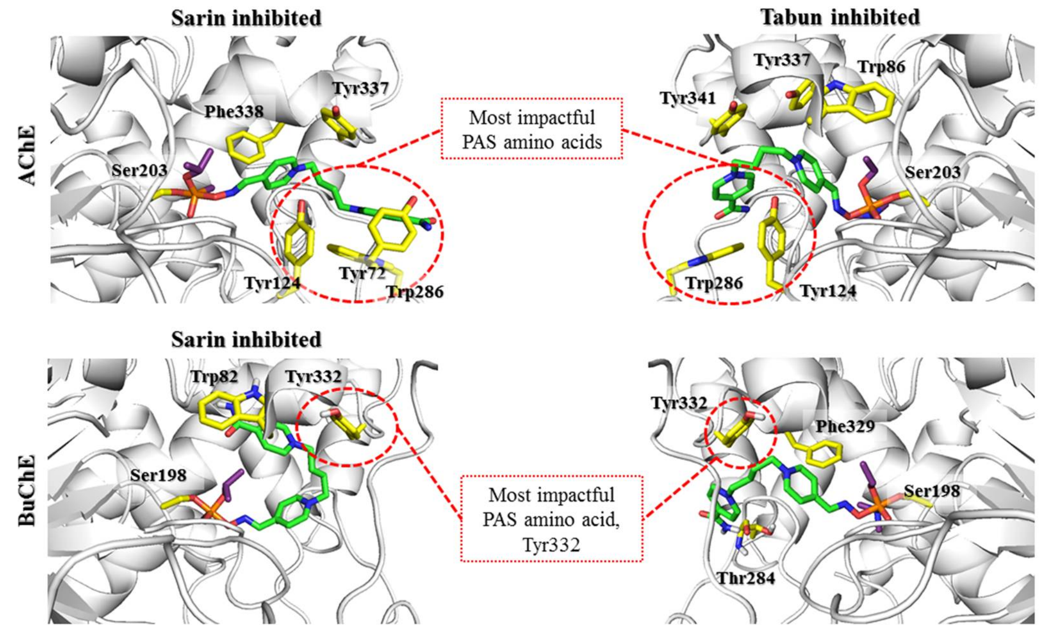

Figure S12. Docking results reflecting the formation of the transitional state by the K203 (green) at the active sites of AChE and BuChE (gray) blocked by sarin and tabun (purple). The key amino acids involved in the interactions with the reactivator are marked with yellow sticks. The interactions of the reactivator with AChE PAS region, observed in the prereactivation state, were further enhanced during the formation of the transitional state. In the case of BuChE, the weakening of the interaction with Tyr332 and Asp70 in the transitional state is not compensated by the PAS amino acids.

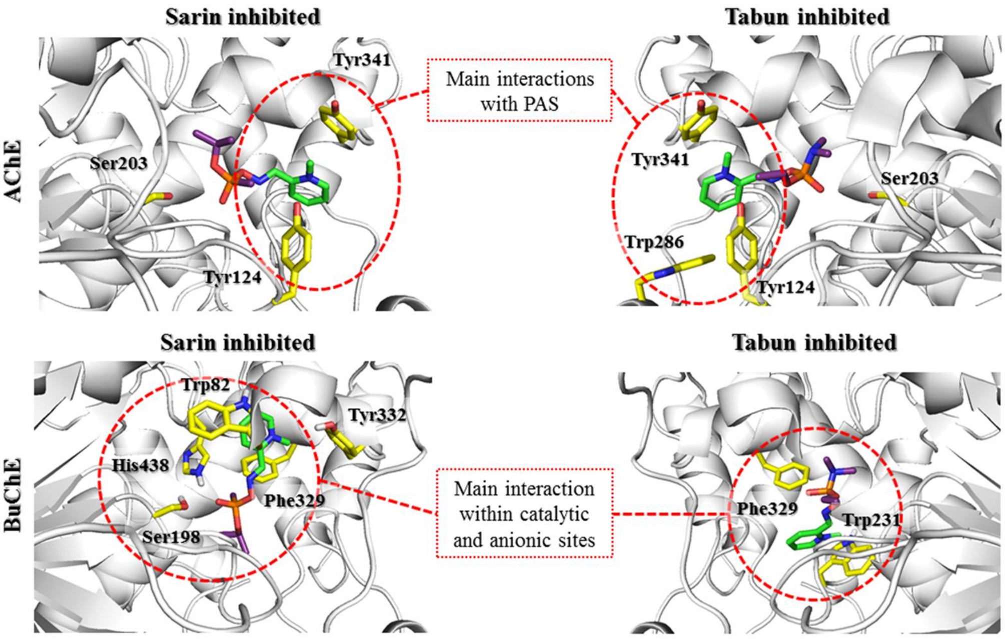

Figure S13. Docking results reflecting the behavior of the created sarin-2-PAM and tabun-2-PAM conglomerates (green) after reactivation of the active sites of AChE and BuChE (gray). The key amino acids involved in the interactions with the OP-reactivator conglomerate are marked with yellow sticks. The lack of aromatic residues within PAS makes it difficult for the postreactivation complex to leave the active site of BuChE.

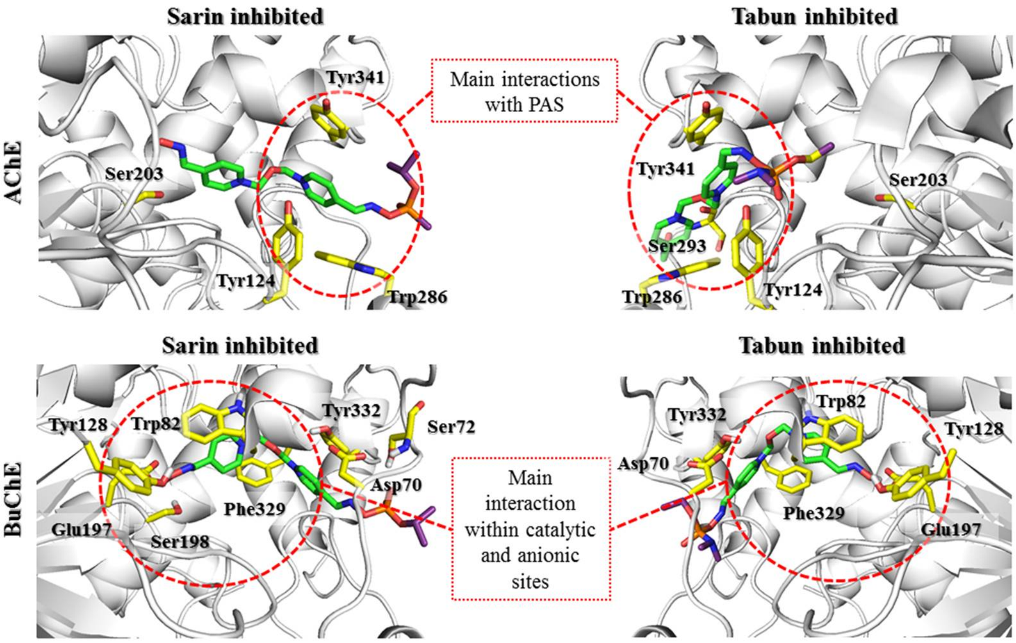

Figure S14. Docking results reflecting the behavior of the created sarin-obidoxime and tabun-obidoxime conglomerates (green) after reactivation of the active sites of AChE and BuChE (gray). The key amino acids involved in the interactions with the OP-reactivator conglomerate are marked with yellow sticks. The lack of aromatic residues within PAS makes it difficult for the postreactivation complex to leave the active site of BuChE.

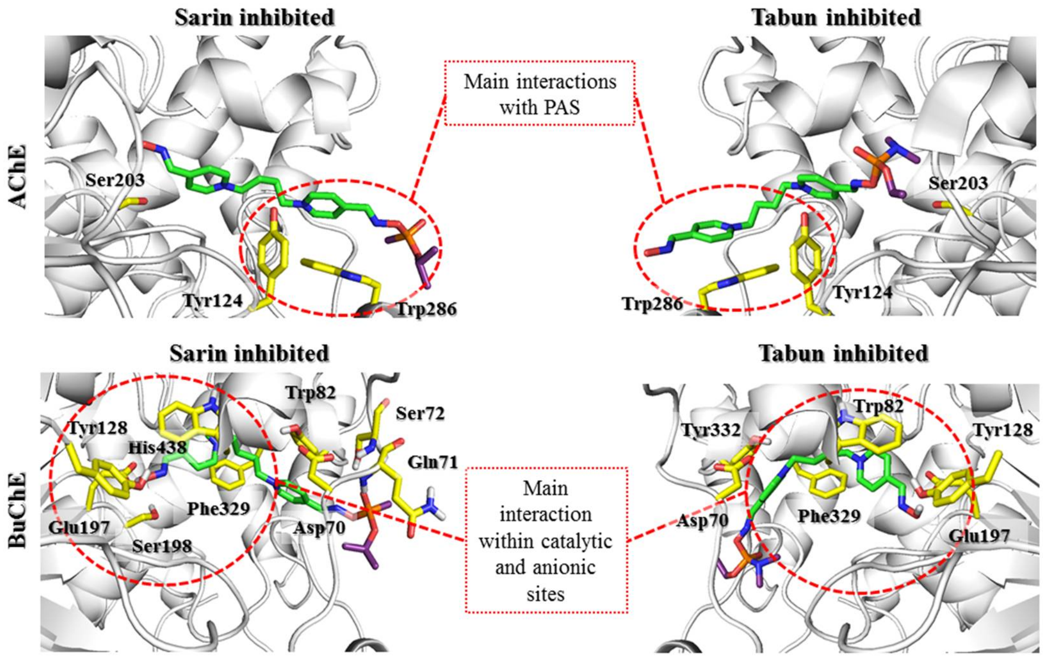

Figure S15. Docking results reflecting the behavior of the created sarin-K074 and tabun-K074 conglomerates (green) after reactivation of the active sites of AChE and BuChE (gray). The key amino acids involved in the interactions with the OP-reactivator conglomerate are marked with yellow sticks. The postreactivation complex binds more strongly at the active site of BuChE than AChE.

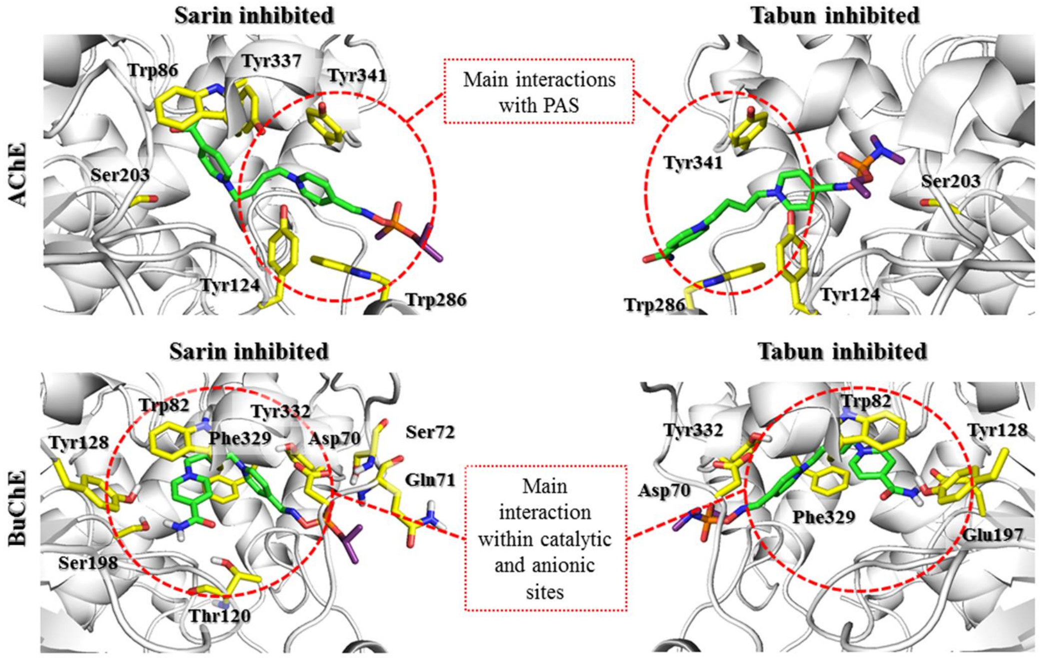

Figure S16. Docking results reflecting the behavior of the created sarin-K203 and tabun-K203 conglomerates (green) after reactivation of the active sites of AChE and BuChE (gray). The key amino acids involved in the interactions with the OP-reactivator conglomerate are marked with yellow sticks. The lack of aromatic residues within PAS makes it difficult for the postreactivation complex to leave the active site of BuChE.
